# Evolution of bismuth-based metal–organic frameworks for efficient electroreduction of CO_2_†

**DOI:** 10.1039/d2ta04485d

**Published:** 2022-08-18

**Authors:** Lili Li, Xinchen Kang, Meng He, Alena Sheveleva, Kui Hu, Shaojun Xu, Yiqi Zhou, Jin Chen, Sergei Sapchenko, George Whitehead, Iñigo J. Vitorica-Yrezabal, Laura Lopez-Odriozola, Louise S. Natrajan, Eric J. L. McInnes, Martin Schröder, Sihai Yang, Floriana Tuna

**Affiliations:** Department of Chemistry, University of Manchester Manchester M13 9PL UK M.Schroder@manchester.ac.uk Sihai.Yang@manchester.ac.uk Floriana.Tuna@manchester.ac.uk; Beijing National Laboratory for Molecular Sciences, CAS Key Laboratory of Colloid, Interface and Chemical Thermodynamics, Institute of Chemistry, Chinese Academy of Science Beijing 100190 China; Photon Science Institute, University of Manchester Manchester M13 9PL UK; UK Catalysis Hub, Research Complex at Harwell Didcot OX11 0FA UK; Cardiff Catalysis Institute, School of Chemistry, Cardiff University Cardiff CF10 3AT UK; Department of Materials, University of Manchester Manchester M13 9PL UK; Institute for Advanced Materials and Technology, University of Science and Technology Beijing Beijing 100083 China

## Abstract

Understanding the structural and chemical changes that reactive metal–organic frameworks (MOFs) undergo is crucial for the development of new efficient catalysts for electrochemical reduction of CO_2_. Here, we describe three Bi(iii) materials, MFM-220, MFM-221 and MFM-222, which are constructed from the same ligand (biphenyl-3,3′,5,5′-tetracarboxylic acid) but which show distinct porosity with solvent-accessible voids of 49.6%, 33.6% and 0%, respectively. We report the first study of the impact of porosity of MOFs on their evolution as electrocatalysts. A Faradaic efficiency of 90.4% at −1.1 V *vs.* RHE (reversible hydrogen electrode) is observed for formate production over an electrode decorated with MFM-220-p, formed from MFM-220 on application of an external potential in the presence of 0.1 M KHCO_3_ electrolyte. *In situ* electron paramagnetic resonance spectroscopy confirms the presence of ·COOH radicals as a reaction intermediate, with an observed stable and consistent Faradaic efficiency and current density for production of formate by electrolysis over 5 h. This study emphasises the significant role of porosity of MOFs as they react and evolve during electroreduction of CO_2_ to generate value-added chemicals.

## Abstract 2

The impact of porosity of MOFs on their structural evolution during reaction with electrolyte and their use as pre-catalysts for electrochemical reduction of CO_2_ provide new insights into the design of new MOFs-derived catalysts.

## Graphical abstract

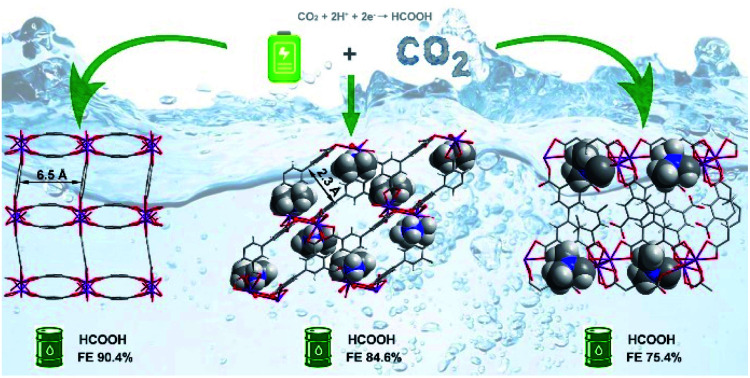

## Introduction

Electrochemical reduction of CO_2_ into fuels and chemical feedstocks enables the storage of renewable electrical energy and is a highly desirable process for carbon neutrality.^1–5^ Formate (or formic acid) has a wide range of applications in industry as a preserving, antibacterial agent as well as liquid fuel, and over one million tonnes of formate is produced annually *via* carbonylation of methanol.^6,7^ The electroreduction of CO_2_ to formate has attracted much attention, and non-precious metals such as Sn, Co, In, Tl, Cd, Hg show catalytic activity for this process.^8–12^ However, these metals generally exhibit drawbacks, such as high cost, toxicity, and low catalytic selectivity. Bismuth is a relatively benign main group metal environmentally^13–15^ and tends to show a poor activity for hydrogen evolution reaction (HER),^16^ which is the main competitive side-reaction during the electrochemical CO_2_ reduction reaction (CO_2_RR). Suppressing the HER will greatly increase the Faradaic efficiency (FE) for the formation of carbon-based products.

Porous metal–organic frameworks (MOFs) have emerged as efficient catalysts for the CO_2_RR owing to their atomically dispersed metal sites and porous structure.^2^ Bismuth-based MOFs are reported to undergo structural evolution to afford active catalysts (primarily Bi nanosheets and particles) during electrochemical reduction of CO_2_.^17–21^ Monitoring the evolution of these Bi materials and exploring the catalytic activity of the resultant materials for CO_2_RR are important to the discovery of efficient new electrocatalysts. Previous studies report various Bi-MOFs constructed from different bridging ligands, but the impact of their porosity on their structural evolution under electrochemical conditions remains largely unexplored.

Herein, we report a comprehensive study of the structural evolution during the CO_2_RR of three Bi-MOFs, namely MFM-220, MFM-221 and MFM-222, constructed from the same ligand (biphenyl-3,3′,5,5′-tetracarboxylic acid, H_4_L). The MOFs were synthesised by solvothermal reactions of Bi(NO_3_)_3_·5H_2_O and H_4_L under different conditions and they show distinct porosity (solvent-accessible void ranging 49.6% to 0%). Powder X-ray diffraction (PXRD), infrared (IR) and Raman spectroscopy, X-ray photoelectron spectroscopy (XPS), and scanning electron microscopy (SEM) have been used to characterise the structural evolution of these Bi-MOFs upon reaction with the electrolyte and on application of an external potential; the new materials are denoted as MFM-200/-221/-222-e (e = electrolyte) and MFM-200/-221/-222-p (p = potential), respectively. MFM-220-p, derived from MFM-220 with the highest porosity, shows the best catalytic performance for CO_2_RR compared with MFM-221-p and MFM-222-p. Thus, in 0.1 M KHCO_3_ electrolyte solution, a total current density of 23 mA cm^−2^ at −1.1 V *vs.* RHE (RHE = reversible hydrogen electrode) and FE_formate_ of 90.4% are observed using an electrode decorated with evolved MFM-220-p. Moreover, MFM-220-p remains active for the CO_2_RR for at least 5 h. Electron paramagnetic resonance (EPR) spectroscopy using the spin trap, 5,5-dimethyl-1-pyrroline-*N*-oxide (DMPO) reveals the presence of ·COOH radicals as a reaction intermediate and rationalises the observed high catalytic stability. This study demonstrates the important role of porosity of MOFs on their evolution to active electrocatalysts for CO_2_RR.

## Results and discussion

**Fig. 1:**
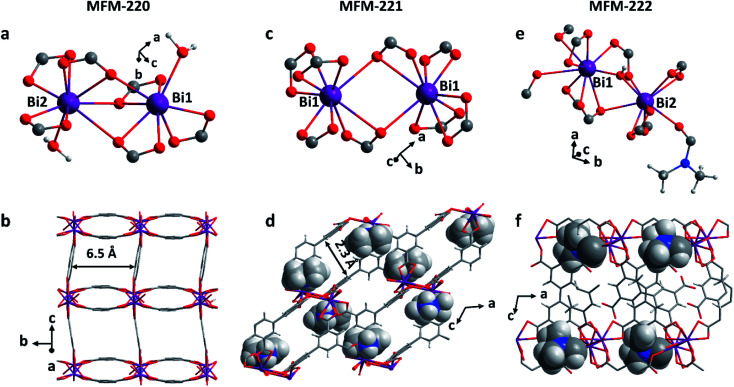
Views of the coordination environments of Bi(iii) sites and pore structures of (a and b) MFM-220, (c and d) MFM-221 and (e and f) MFM-222 (Bi, purple; O, red; C, grey; H, light grey; N, blue). The Me_2_NH_2_^+^ cations and coordinated DMF molecules in MFM-221 and MFM-222, respectively, are highlighted in ball-and-stick mode.

Solvothermal reactions of Bi(NO_3_)_3_·5H_2_O and H_4_L in CH_3_CN and dimethylformamide (DMF) with different amounts of HNO_3_ (5%) and different reaction times (see ESI† for details) afford single crystals of MFM-220 {[Bi_2_(L)_1.5_(H_2_O)_2_]·3.5DMF·3H_2_O}, MFM-221 {[Bi(L)]·Me_2_NH_2_·1.5DMF} and MFM-222 {[Bi_2_(HL)·(H_2_L)·(DMF)·(OH)]} in pure phase (Fig. S1†). MFM-220, MFM-221 and MFM-222 all crystallize in monoclinic systems (Table S1†). We have previously reported^22^ MFM-220, which shows a non-interpenetrated neutral framework structure constructed from binuclear {Bi_2_} centres bridged by tetracarboxylate ligands (Fig. 1a and b). MFM-220 co-crystallizes in α- and β-forms with slightly altered coordination environments at the Bi(iii) sites (differing only in the coordination of a H_2_O molecule) but with the same pore size and adsorption properties. In both phases, each Bi(iii) ion is coordinated to three carboxylate groups from three different L^4−^ ligands, and the two Bi(iii) ions in the binuclear {Bi_2_} centres share three coordinated oxygen atoms from three bridging carboxylate groups. In α-MFM-220, each Bi(iii) ion also coordinates to one terminal H_2_O to give a coordination number of 8 for Bi1 and 9 for Bi2 (Fig. 1a). MFM-220 displays micropores with a pore size of 6.5 Å, and the accessible solvent voids is 49.6% calculated by Platon (Fig. 1b, S2a and S4†).^23^ The synthesis and structural characteristics of MFM-221 and MFM-222 have not been reported previously. MFM-221 forms an anionic non-interpenetrated framework where the secondary building units are formed by binuclear {Bi_2_} moieties. Each Bi(iii) ion binds to five ligands with four chelating carboxylates and one in monodentate mode, and adjacent Bi(iii) ions are bridged by two carboxylates, giving a coordination number of 9 (Fig. 1c). The pore is occupied by counter-cations Me_2_NH_2_^+^ and uncoordinated DMF molecules (Fig. S2b and S5†), the Me_2_NH_2_^+^ cations being generated by *in situ* decompositions of the DMF solvent during the reaction. MFM-221 becomes porous after removing free DMF molecules, resulting in a solvent-accessible volume of 33.6% calculated by Platon (Fig. 1d).^23^ MFM-222 has a neutral, non-interpenetrated and non-porous structure constructed from binuclear {Bi_2_} centres bridged by tetracarboxylate ligands. The Bi1 and Bi2 centres share one carboxylate oxygen from a ligand and a bridging hydroxyl group (μ_2_-OH). Bi1 is coordinated to six ligands, two in bidentate and four in monodentate mode. Bi2 is coordinated to five ligands, two in bidentate and three in monodentate mode (Fig. 1e). Bi2 also has a bound DMF molecule giving an overall non-porous structure for MFM-222 (Fig. 1f, S2c and S6†). The purity of bulk materials of MFM-220, MFM-221 and MFM-222 (Fig. S7†) was confirmed by PXRD (Fig. S8†).

**Fig. 2:**
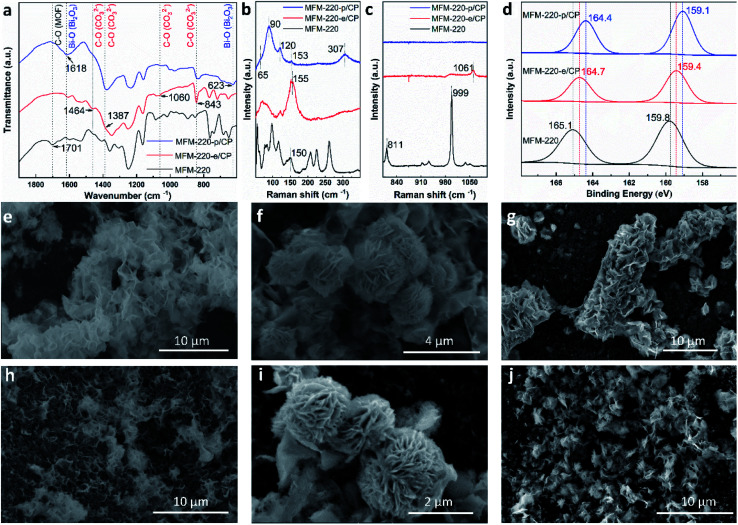
Characterisation of the transformation and evolution of Bi-MOFs to Bi_2_O_2_CO_3_ nanosheets and α-Bi_2_O_3_. (a) FT-IR spectra; (b and c) Raman spectra; (d) XPS spectra; SEM images of (e) MFM-220-e/CP, (f) MFM-221-e/CP, (g) MFM-222-e/CP, (h) MFM-220-p/CP, (i) MFM-221-p/CP, and (j) MFM-222-p/CP.

MFM-220, MFM-221 and MFM-222 were used as precursors to prepare active catalysts for CO_2_RR. Carbon paper (CP) was used as the substrate for the preparation of working electrodes. Electrochemical CO_2_RR was conducted in an H-type cell with a three-electrode configuration (Fig. S9†). Both the catholyte and anolyte were 0.1 M KHCO_3_ aqueous solutions separated by a Nafion-117 membrane. Before conducting electrocatalysis, we studied the reactivity of these Bi materials in the electrolyte solution only in the absence an external potential. The as-prepared MOF/CP electrodes were placed into the electrolyte for 30 minutes to afford MFM-220-e/CP, MFM-221-e/CP and MFM-222-e/CP. PXRD patterns suggest the disappearance of the Bragg peaks of the pristine MOFs and new peaks assigned to Bi_2_O_2_CO_3_ become evident (Fig. S10†).^21^ While porous MFM-220 and MFM-221 show complete structural transition to Bi_2_O_2_CO_3_, MFM-222-e exhibits a mixture of MFM-222 and Bi_2_O_2_CO_3_ owing probably to its non-porous nature (Fig. S10c†). IR, Raman and XPS analyses were used to characterise the resultant materials supported on CP. The IR spectrum of MFM-220-e/CP confirms the absence of characteristic bands for the ligands, whilst new bands at 1464, 1387, 1060 and 843 cm^−1^ are assigned to the carbonate groups in Bi_2_O_2_CO_3_ (Fig. 2a).^24,25^ Comparison of the Raman spectra of MFM-220 and MFM-220-e/CP revealed (i) the disappearance of bands at 811 and 999 cm^−1^ (assigned to the C–H and C=C groups in the ligands), (ii) a shift in the Bi–O vibrational band from 150 to 155 cm^−1^, and (iii) the appearance of a new band at 1061 cm^−1^ (assigned to the stretching vibration of C–O in Bi_2_O_2_CO_3_), fully consistent with the structural transition to Bi_2_O_2_CO_3_ (Fig. 2b, c and S11†).^25–27^ The XPS spectrum of MFM-220 shows two peaks of Bi 4f at 165.1 and 159.8 eV, consistent with the expected Bi(iii) centers within the material (Fig. 2d). In MFM-200-e/CP, these peaks move to slightly lower binding energy at 164.7 and 159.4 eV, again consistent with a Bi(iii) in Bi_2_O_2_CO_3_.^28^ Similar IR, Raman and XPS results are found for MFM-221-e/CP and MFM-222-e/CP, except that an incomplete structural transition is observed for MFM-222-e (Fig. S11–S14†). Raman spectroscopy as a function of time confirms that MFM-220 undergoes a more rapid structural transition than MFM-221 or MFM-222 upon soaking in electrolyte. MFM-220 and MFM-221 start to evolve after 5 and 10 min, respectively, while MFM-222 shows retention of characteristic Raman features even after 20 min (Fig. S12a–c†).

Further structural evolution to MOF-e/CP electrodes occurred upon application of an external potential to give MFM-220-p/CP, MFM-221-p/CP and MFM-222-p/CP. The PXRD patterns of these transformed species show characteristic peaks assigned to α-Bi_2_O_3_ with minor amounts of metallic Bi (Fig. S10d–f†). IR, Raman and XPS spectra verified the formation of α-Bi_2_O_3_ and Bi (Fig. 2).^29^ For example, Raman spectra of MFM-220-p/CP show the characteristic bands of α-Bi_2_O_3_ at 120, 153 and 307 cm^−1^ (Fig. 2b).^25,30^ An additional two new Raman peaks appear at 65 and 90 cm^−1^ are assigned to the *E*_g_ and *A*_1g_ stretching modes of Bi–Bi bonds, respectively.^31–33^ The XPS spectra of MFM-220-p/CP show further slight shifts of the 4f peaks for Bi (164.4 and 159.1 eV) to the low energy region compared with MFM-220-e/CP (164.7 and 159.4 eV), indicating the partial reduction of Bi(iii) to Bi(0).^20^ Thus, α-Bi_2_O_3_ and Bi co-exist in MFM-220-p/CP. Similar IR, Raman and XPS results are observed for MFM-221-p/CP (Fig. S11–S14†). In contrast, MFM-222-p/CP retains Raman bands at 1052 cm^−1^ and the IR band at 844 cm^−1^ (Fig. S11f and S13b†), indicating the presence of a mixture of Bi_2_O_2_CO_3_, α-Bi_2_O_3_ and Bi. Analysis of the time-resolved Raman spectra shows that the structural evolution of MFM-220-e and MFM-221-e is completed in 10 and 30 min, respectively, while that for MFM-222-e is not completed even after 50 min (Fig. S12d–f†). The SEM images confirm that pristine MFM-220, MFM-221 and MFM-222 all show well-defined single crystals (Fig. S15†). During the first stage evolution, the morphologies of MFM-220-e/CP, MFM-221-e/CP and MFM-222-e/CP turn into nanosheets (Fig. 2e–g). Little further change to the morphology was observed upon applying the potential (Fig. 2h and i). The SEM-EDX analysis shows the homogeneous distribution of Bi and O in the resulting materials during the two-stage structural evolution process (Fig. S16–S21†). The EPR spectra of MFM-220-p/CP, MFM-221-p/CP and MFM-222-p/CP all show an apparent signal at *g* = 2.0033, which is assigned to the oxygen vacancies in the resulting α-Bi_2_O_3_.^34–36^ Thus, these results confirm that the MOF-p working electrodes are mainly composed of α-Bi_2_O_3_.

**Fig. 3:**
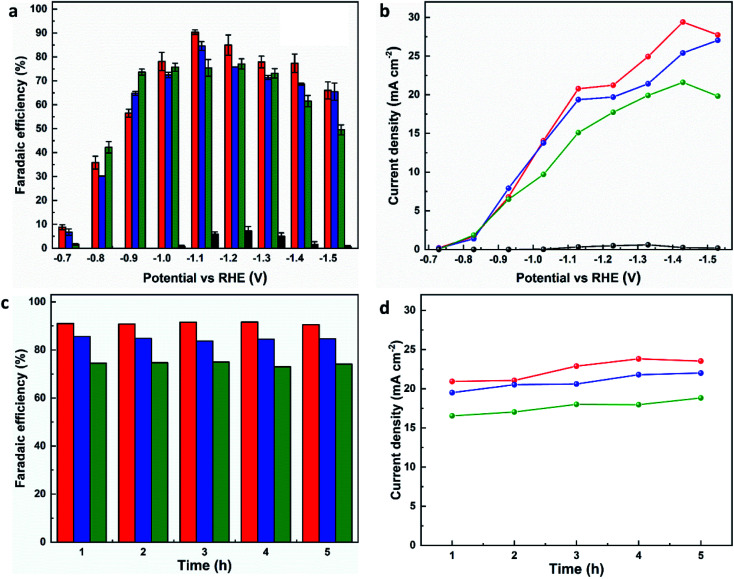
Catalytic performance of MFM-220-p/CP (red lines or column), MFM-221-p/CP (blue lines or column), MFM-222-p/CP (green lines or column), and CP (black lines or column) electrodes for CO_2_RR in 0.1 M KHCO_3_. Plots of (a) FE_formate_*vs.* potential, (b) current density for formate production *vs.* potential, (c) FE_formate_*vs.* time, and (d) current density of formate *vs.* time for reduction of CO_2_ at −1.1 V *vs.* RHE.

The electrocatalytic CO_2_RR performance of the evolved working electrodes was investigated in an H-cell. A bare CP electrode was included for comparison. Formate was found to be the main carbon-containing product by analysing the products in both gas and liquid phases after the electrolysis by GC, FTIR and ^1^H NMR spectroscopy. H_2_ is the only by-product. All three MOF-p/CP electrodes give higher FE_formate_ and current density of formate at all potentials than the bare CP electrode (Fig. 3a and b), with MFM-220-p/CP showing better catalytic performance than MFM-221-p/CP and MFM-222-p/CP. The highest FE_formate_ over MFM-220-p/CP reached 90.4% at −1.1 V *vs.* RHE with a total current density of 23.0 mA cm^−2^ after electrolysis for 1 h. The partial current density of formate over MFM-220-p/CP is also higher than that for MFM-221-p/CP and MFM-222-p/CP (20.8, 19.3 and 15.1 mA cm^−2^, respectively, Fig. 3b). In comparison, the value of FE_formate_ over MFM-221-p/CP and MFM-222-p/CP electrodes are 84.6% and 75.4%, respectively (Fig. 3a). The enhanced catalytic performance of MFM-220-p/CP compared with the cation-blocked MFM-221 and non-porous MFM-222 is attributed to its structural evolution promoted by its highly porous structure. The slow evolution of non-porous MFM-222 and the presence of Bi_2_O_2_CO_3_ in MFM-222-p/CP result in reduced active sites for CO_2_RR, leading to lower catalytic activity (Fig. S14b†).

To confirm that CO_2_ is the sole carbon source for producing formate, a control experiment was conducted in an Ar-saturated electrolyte without CO_2_, and no formate was detected (Fig. S26†). The change of FE_formate_ and current density with time were recorded at −1.1 V *vs.* RHE using all three MOF-p/CP electrodes. All three electrodes show stable FE_formate_ and the current density of formate for the electrolysis over at least 5 h (Fig. 3c and d). The overall catalytic performance of MFM-220-p/CP is comparable with the leading Bi-based catalysts studied for CO_2_RR in an H-cell (Fig. S27 and Table S2†).

**Fig. 4:**
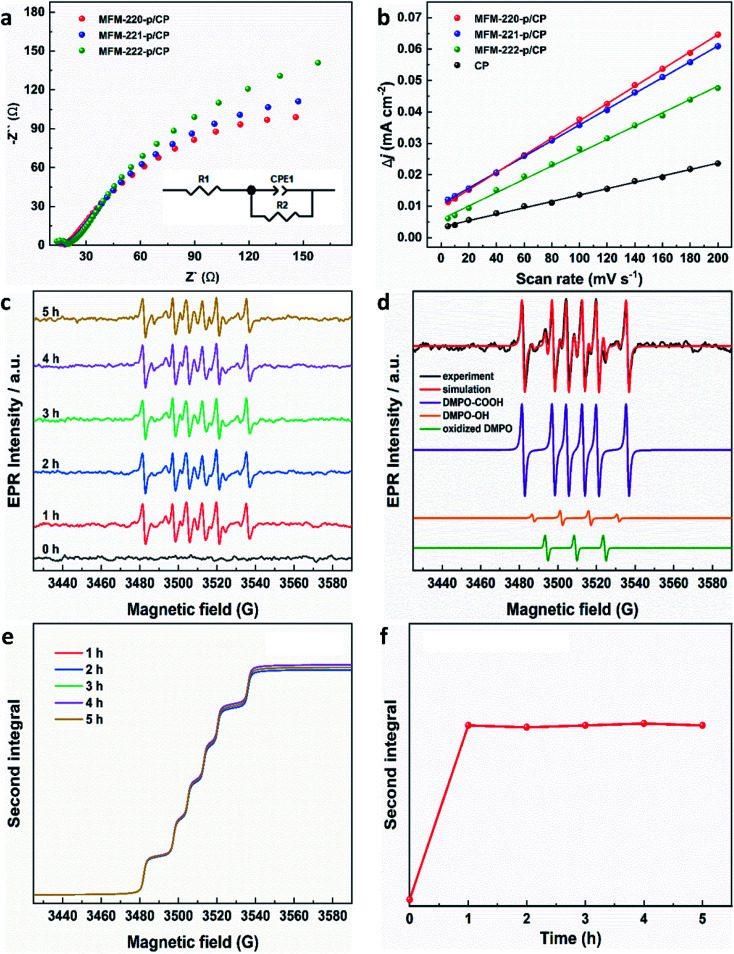
Electrochemical characterization of reconstructed working electrodes: (a) EIS spectra (the inset is the corresponding equivalent circuit); (b) plot of difference in charging current density *vs.* scan rates. EPR characterization of radicals produced during CO_2_RR using MFM-220-p/CP at −1.1 V *vs.* RHE: (c) EPR spectra of aliquots of electrolyte taken at different times; (d) EPR spectra of spin adducts of free radicals observed. The complete set of parameters for simulations are given in Table S3;† (e) second integrals of simulated X-band EPR spectra for DMPO-·COOH adduct *vs.* time; (f) plot of the second integral of the X-band EPR signals for DMPO-·COOH at room temperature *vs.* time.

Electrochemical impedance spectroscopy (EIS) and the electrochemical active surface area (ECSA) were studied to elucidate the electrochemical activity of these electrodes using the same set-up as above. The charge-transfer resistance of three evolved MOF electrodes was revealed by EIS spectra to characterize the charge exchange between the catalyst and reactant in the electrolyte. As shown in Fig. 4a, MFM-220-p/CP, MFM-221-p/CP and MFM-222-p/CP show a resistance (*R*_ct_) to charge transfer of 329.8, 397.6 and 456.8 Ω cm^2^, respectively. The rapid and thorough transformation of porous MFM-220 results in enhanced conductivity for MFM-220-p/CP, thus promoting its performance for CO_2_RR. The double-layer capacitance (*C*_dl_) for all three reconstructed MOF electrodes was analyzed by measuring the capacitive current associated with double-layer charging using the scan-rate dependence of cyclic voltammetric stripping.^9^ MFM-220-p/CP has the highest value for *C*_dl_ at 0.2743 mF cm^−2^, and MFM-221-p/CP, MFM-222-p/CP and CP show values of 0.2511, 0.2119 and 0.1016 mF cm^−2^, respectively (Fig. 4b). This again is consistent with the observed high catalytic activity of MFM-220-p/CP.

EPR spectroscopy was employed to monitor and characterize as a function of time any intermediate radicals produced during the electroreduction process.^37^ EPR spectra were measured for aliquots of electrolyte solution taken at time intervals of 1 h during reduction of CO_2_ at −1.1 V *vs.* RHE over all three MOF-p/CP electrodes. DMPO was used as a spin trapping agent to identify the short-lived radicals during CO_2_RR.^38^ Characteristic spectra of DMPO-adduct radicals were observed for all three electrodes (Fig. 4c, S31 and S32†). The spectra are dominated by a six-line pattern consistent with the DMPO-COOH adduct (hyperfine coupling constants *a*_N_ = 15.6 G, *a*_H_ = 22.9 G), with minor quantities of an oxidized DMPO radical (*a*_N_ = 15.1 G) and the DMPO-OH adduct (*a*_N_ = 14.7 G, *a*_H_ = 14.7 G; simulations in Fig. 4d, with parameters in Table S3†).^38–40^ This is consistent with formate being the only carbon-containing product in this reaction and also established pathways for CO_2_ reduction *via*
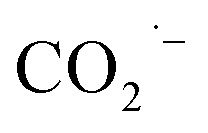
 and/or ·COOH radicals.^41–43^ The hyperfine coupling constants are more consistent with trapped ·COOH radicals rather than trapped 
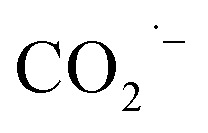
.^38^ To ensure that the ·COOH radicals come from CO_2_RR, and not the electrolyte, we also performed the EPR measurements with 0.1 M KHCO_3_ and 0.1 M KOH as the reference, in the absence of CO_2_. No EPR signals were detected, confirming that ·COOH is exclusively produced in the presence of CO_2_ (Fig. S31c†). To monitor the production of ·COOH during electrolysis on three evolved MOF-p/CP electrodes as a function of time, spectra were taken from aliquots at 1 h intervals over a 5 h electrolysis. Analysis of the second integral of the EPR signals (Fig. 4e and f, S32–S34†) shows that the radicals are being formed continuously and at a similar rate (with a continuous supply of CO_2_) throughout this timescale. This is consistent with the observed stable Faradaic efficiency and current density for formate production over this timescale (Fig. 3c and d).

In summary, three Bi-MOFs with the same ligand but distinct porosity (ranging from 49.6% to 0%) have been synthesised by tuning the reaction conditions. PXRD, IR, Raman, XPS and SEM-EDX have been used to characterise the structural evolution of these Bi-MOFs upon reacting with electrolyte and applying the external potential. A value for FE_formate_ can reach 90.4% at −1.1 V *vs.* RHE using evolved MFM-220-p/CP. The high catalytic ability of MFM-220-p/CP is due to the structural evolution promoted by the highly porous structure of MFM-220 (void of 49.6%) compared to the cation-blocked MFM-221 and non-porous MFM-222 (voids of 33.6% and 0%, respectively). EPR spectroscopy identified the formation of ·COOH as a key radical reaction intermediate in this system, and confirmed that the generation of ·COOH radical remained constant during the CO_2_RR over at least 5 h. This study emphasises the significant impact of the porosity of MOFs on their evolution during the electrochemical CO_2_RR process.

## Conflicts of interest

The authors declare no competing financial interest.

## Supplementary Material

TA-010-D2TA04485D-s001

TA-010-D2TA04485D-s002

TA-010-D2TA04485D-s003
