# Supplementary material for: Evolution of bismuth-based metal–organic frameworks for efficient electroreduction of CO2
Source: J Mater Chem A Mater. 2022 Aug 18;10(34):17801–7. doi: 10.1039/d2ta04485d (PMC9426795; doi:10.1039/d2ta04485d)
Supplement: TA-010-D2TA04485D-s001 [file TA-010-D2TA04485D-s001.pdf]

Electronic Supplementary Material (ESI) for Journal of Materials Chemistry A.
This journal is © The Royal Society of Chemistry 2022

Supplementary Information

Evolution of bismuth-based metal-organic frameworks for efficient electroreduction of CO_2

Lili Li[1], Xinchen Kang[1,2], Meng He[1], Alena Sheveleva[1,3], Kui Hu[1], Shaojun Xu[4,5], Yiqi Zhou[6,7], Jin Chen[1], Sergei Sapchenko[1], George Whitehead[1], Iñigo J. Vitorica-Yrezabal[1], Laura Lopez-Odriozola[1], Louise S. Natrajan[1], Eric J. L McInnes[1,2], Martin Schröder[1]*, Sihai Yang[1]* and Floriana Tuna[1,2]*

1. Department of Chemistry, University of Manchester, Manchester, M13 9PL (UK)

2. Beijing National Laboratory for Molecular Sciences, CAS Key Laboratory of Colloid, Interface and Chemical Thermodynamics, Institute of Chemistry, Chinese Academy of Science, Beijing, 100190 (China)

3. Photon Science Institute, University of Manchester, Manchester, M13 9PL (UK)

4 UK Catalysis Hub, Research Complex at Harwell, Didcot, OX11 0FA, UK

5 Cardiff Catalysis Institute, School of Chemistry, Cardiff University, Cardiff, CF10 3AT, UK

6. Department of Materials, University of Manchester, Manchester, M13 9PL (UK)

7. Institute for Advanced Materials and Technology, University of Science and Technology Beijing, Beijing 100083 (China)

EXPERIMENTAL SECTION

Materials. $Bi(NO_3)_3 \cdot 5H_2O$ (>99%), nitric acid (70%), acetonitrile (CH_3CN) (>99%), dimethylformamide (DMF) (>99%), acetone (>99%), piperazine (>99%), dimethyl sulfoxide(DMSO), 5,5-dimethyl-1-pyrroline-N-Oxide (DMPO) and deuterium oxide (D_2O) were purchased from Sigma-Aldrich Co., UK. Carbon paper (CP) was purchased from Gaoss Union. $KHCO_3$ (99%), Nafion D521 solution (5 % w/w in water and 1-propanol, ≥ 0.92 meg/g exchange capacity) and Nafion N-117 membrane (0.180 mm thick, ≥ 0.90 meg/g exchange capacity) were purchased from Alfa Aesar Co., UK. Biphenyl-3,3',5,5'-tetracarboxylic acid (H_4L) was synthesised according to the literature procedure.[1] All chemicals and reagents used in this study were used as received without further purification.

Synthesis of single crystals of MFM-220, MFM-221 and MFM-222. The reaction conditions are shown in Figure S1 and the crystallographic data are summarised in Table S1.

Synthesis of $[Bi_2(C_{16}H_6O_8)_{1.5}(H_2O)_2] \cdot 3.5DMF \cdot 3H_2O$ (MFM-220). H_4L (15 mg, 0.045 mmol), $Bi(NO_3)_3 \cdot 5H_2O$ (17 mg, 0.035 mmol) and piperazine (7 mg, 0.081 mmol) were dispersed in a mixture of DMF and MeCN (1.3 mL, 1:0.3 v/v). 5% HNO_3 (0.3 mL) was added to the white slurry, which was transferred into a 23 mL glass pressure tube. The vial was sealed and heated at 100 °C in an oil bath for 10 h. The product was collected, washed five times with DMF and dried briefly in air. Yield: 10 mg (25 %). Elemental analysis (% calc/found): $[Bi_2(C_{16}H_6O_8)_{1.5}(H_2O)_2] \cdot 3.5DMF \cdot 3H_2O$ (C 34.5/34.0, H 3.6/3.1, N 3.5/4.1).

Synthesis of $[Bi(C_{16}O_8H_6)] \cdot C_2H_8N \cdot 1.5DMF$ (MFM-221). H_4L (15 mg, 0.045 mmol), $Bi(NO_3)_3 \cdot 5H_2O$ (17 mg, 0.035 mmol) and piperazine (7 mg, 0.081 mmol) were dispersed in a mixture of DMF and MeCN mixture (1.3 mL, 1:0.3 v/v). 5% HNO_3 (0.3 mL) was added to the resulting white slurry, which was transferred into an 8 mL glass vial. The vial was heated at 100 °C for 9 days. The product was collected, washed five times with DMF and used for single-crystal XRD characterisation. Yield: 8.0 mg (20 %). Elemental analysis (% calc/found): $[Bi(C_{16}O_8H_6)] \cdot C_2H_8N \cdot 1.5DMF$ (C 39.0/38.6, H 3.5/3.7, N 5.0/5.3).

Synthesis of $[Bi_2(C_{16}O_8H_7) \cdot (C_{16}O_8H_8) \cdot (DMF) \cdot (OH)]$ (MFM-222). H_4L (15 mg, 0.045 mmol), $Bi(NO_3)_3 \cdot 5H_2O$ (17 mg, 0.035 mmol) and piperazine (7 mg, 0.081 mmol) were dispersed in the mixture of DMF and MeCN (1.3 mL, 1:0.3 v/v). 5% HNO_3 (0.8 mL) was added to the resultant white slurry, which was transferred into an 8 mL glass vial. The vial was heated at 100 °C for 4 days. The product was collected, washed five times with DMF and used for single-crystal XRD characterisation. Yield: 10 mg (25 %). Elemental analysis (% calc/found): $[Bi_2(C_{16}O_8H_7) \cdot (C_{16}O_8H_8) \cdot (DMF) \cdot (OH)]$ (C 36.0/35.7, H 1.97/1.92, N 1.20/1.18).

Synthesis of powder samples of MFM-220, MFM-221 and MFM-222. The reaction schemes are presented in Figure S7.

Synthesis of MFM-220. H_4L (60 mg, 0.180 mmol), $Bi(NO_3)_3 \cdot 5H_2O$ (68 mg, 0.140 mmol) and piperazine (28 mg, 0.325mmol) were combined in a 40 mL microwave vial, and DMF (4 mL), CH_3CN (1.2 mL) and 5% HNO_3 (1.2 mL) were added. The vial was placed into the microwave reactor and heated at 100 ^oC for 2 h. The resultant powder was collected and washed several times with DMF and acetone and dried. Yield: 57 mg (65 %). Elemental analysis (% calc/found): $[Bi_2(C_{16}H_6O_8)_{1.5}(H_2O)_2]\cdot 3.5DMF\cdot 3H_2O$ (C 34.5/34.3, H 3.6/3.3, N 3.5/3.8).

Synthesis of MFM-221. H_4L (30 mg, 0.090 mmol), $Bi(NO_3)_3 \cdot 5H_2O$ (34 mg, 0.070 mmol) and piperazine (14 mg, 0.163 mmol) were combined in a 20 mL glass vial, and DMF (2 mL), CH_3CN (0.6 mL) and 5% HNO_3 (0.6 mL) were added. The vial was heated at 120 ^oC in an oven for 24 h. The resultant powder was collected and washed several times with DMF and dried. Yield: 26 mg (53 %). Elemental analysis (% calc/found): $[Bi(C_{16}O_8H_6)]\cdot C_2H_8N\cdot 1.5DMF$ (C 39.0/39.3, H 3.5/3.6, N 5.0/5.2).

Synthesis of MFM-222. H_4L (60 mg, 0.180 mmol), $Bi(NO_3)_3 \cdot 5H_2O$ (68 mg, 0.140 mmol) and piperazine (28 mg, 0.325mmol) were combined in a 40 mL microwave vial, and DMF (4 mL), acetonitrile (1.2 mL) and 5% HNO_3 (3.2 mL) were added. The vial was placed into the microwave reactor and heated at 130 ^oC for 5 h. The resultant powder was isolated and washed several times with DMF and dried. Yield: 45 mg (56 %). Elemental analysis (% calc/found): $[Bi_2(C_{16}O_8H_7)\cdot(C_{16}O_8H_8)\cdot(DMF)\cdot(OH)]$ (C 36.0/35.4, H 1.97/1.94, N 1.20/1.17).

Material characterisation. PXRD data were collected from a Philips X'pert X-ray diffractometer (45 kV and 40 mA) using Cu- Kα radiation (λ = 1.5406 Å), and the scan speed was 1°/min. Fourier Transform Infrared (FTIR) spectra were recorded on a Nicolet iS5 spectrometer, and Raman spectra on a Horiba XploRA Plus Raman microscope with a 785 nm laser with a 1800 mm^{-1} grating. Baseline correction was applied to all Raman spectra. X-ray photoelectron spectrum (XPS) analysis was performed using a Thermo Fisher Scientific NEXSA spectrometer fitted with a mono-chromated Al *Kα* X-ray source (1486.7 eV). Data were recorded at pass energies of 200 eV for survey scans and 50 eV for the high-resolution scan with 1 eV and 0.1 eV step sizes, respectively, at a spot size of approximately 400 μm. The charge neutralization of the sample was achieved using a combination of both low-energy electrons and Ar ions. C 1s electron at 284.8 eV was used as a standard reference to calibrate the photoelectron energy shift. All the data analyses were performed on Casa XPS software (version: 2.3.22PR1.0). The morphologies of the materials were measured by scanning electron microscopy (SEM) on a Quanta FEG 650.

Electrochemical study. All electrochemical experiments were carried out on the electrochemical workstation (CHI 660E, USA) with an H-type cell. The working electrodes MFM-220/CP, MFM-221/CP, and MFM-222/CP were prepared using the following procedure: 10 mg of MFM-220, MFM-221 or MFM-222 was suspended in isopropanol (1 mL) and H_2O (1 mL) containing 10 μL Nafion D-521 dispersion (5 wt%) which

treated with ultrasound for 30 min to form a homogeneous ink. 100 μL of the ink was spread onto the CP (0.5×1 cm^2) surface and dried at room temperature.

An H-type cell with a three electrodes configuration was used for the electrochemical study (Figure S9). This cell is consists of a working electrode, a platinum gauze as the counter electrode, and Ag/AgCl (submerged in saturated KCl) as the reference electrode. Both the catholyte and anolyte were 0.1 M $KHCO_3$, which was separated by the Nafion-117 membrane. CO_2 was bubbled into the catholyte before the experiments for 30 minutes, and CO_2 was continually bubbled into the catholyte during the electrolysis. After the electrolysis, the liquid products were measured by ^1H NMR spectroscopy and the gas products were collected using a gasbag and analysed by GC and a Bruker Matrix MG5 FTIR spectrometer.

The Electrochemical impedance spectroscopy (EIS) was recorded at −0.5 V vs RHE with an amplitude of 5.0 mV (10^{-1} to 10^6 Hz). The value for the resistance of charge transfer (R_{ct}) was obtained by fitting the EIS spectra using the Zview software (Version 3.5f, Scribner Associates, Inc). Linear sweep voltammetry (LSV) scans were conducted in CO_2 and Ar saturated catholyte.

Quantitative analysis of products in liquid and gas phase. All liquid products were quantified by ^1H NMR spectroscopy. DMSO (1g) was dissolved into H_2O (25 mL) and this solution was used as a reference for calculating the Faradaic efficiency (FE) of formate production during the CO_2RR. After CO_2RR, 100 μL of the as-prepared reference solution of DMSO was injected into the catholyte. Then 0.9 mL of catholyte was mixed with 0.1 mL D_2O, and around 0.7 mL of this solution was subsequently transferred into the NMR tube for measurements. The value of FE was calculated using the equation:

$$FE(\%) = \frac{n_{product} \times n_{electrons} \times F}{Q} \times 100\%$$

where $n_{product}$ is the amount of product (mol) from GC, Bruker Matrix MG5 FTIR spectrometer or ^1H NMR spectroscopy (formic acid), $n_{electrons}$ is electron transfer number (both the production of H_2 and formate are two-electron processes), F is the Faraday constant (96485 C mol^{-1}), and Q is the total charge passed during the CO_2RR.

The potential versus Ag/AgCl was converted to the potential versus RHE (RHE = reversible hydrogen electrode) using the following Equation:

$$E\ (V_{RHE}) = E\ (V_{Ag/AgCl}) + 0.197 + 0.059 * pH$$

EPR measurement. CW electron paramagnetic resonance (EPR) spectroscopy was carried out at X-band (9.85 GHz) on a Bruker Micro EPR spectrometer at room temperature with a microwave power of 6.325 mW. EPR spectra were collected with a modulation amplitude of 1 G. DMPO (200 mmol/L) was dissolved in Ar-

degassed deionised water as a spin trap. Electrolysis was carried out at -1.1 V vs RHE for 1h using MFM-220-p/CP, MFM-221-p/CP or MFM-222-p/CP as the working electrode. During electrolysis, a syringe containing 0.1 mL as-prepared DMPO aqueous solution was used to extract 0.2 mL of electrolyte from the H-cell. The mixed liquid was put into a quartz capillary for EPR investigation. Factors such as concentration of DMPO, the position of the EPR tube, the volume of measured solvent were controlled to achieve accurate quantitative measurement of any generated radicals. Strong pitch (g = 2.0028) was used as a reference sample when measuring X-band EPR spectra. Theoretical modelling of EPR spectra was performed using EasySpin toolbox (Version 6.0.0-dev.34)[2] for Matlab.

Crystallographic section

Data Collection. X-Ray data for compounds MFM-221 and MFM-222 were collected at 100 K using Cu-kα radiation on a Rigaku FR-X rotating anode with a Hypix 6000HE detector.

Crystal structure determinations and refinements. X-Ray data were processed and reduced using CrysAlisPro suite of programmes. Absorption correction was performed using empirical methods (SCALE3 ABSPACK) based upon symmetry-equivalent reflections combined with measurements at different azimuthal angles. The crystal structures were solved and refined against all F^2 values using the SHELX and Olex 2 suite of programmes.[3] All atoms were refined anysotropically and hydrogen atoms were placed in the calculated positions. C-O and C-N distances were restrained using same distance SHELX commands (SADI) and be flat using SHELX commands (FLAT). The atomic displacement parameters of both MFM-221 and MFM-222 structures were restrained using similar and rigid body SHELX commands (SIMU and RIGU). Crystal of MFM-222 was found to be a racemic twin.

CCDC 899427 (MFM-220), 2126590 (MFM-221) and 2126600 (MFM-222) contains the supplementary crystallographic data for this paper. These data can be obtained free of charge via www.ccdc.cam.ac.uk/conts/retrieving.html (or from the Cambridge Crystallographic Data Centre, 12 Union Road, Cambridge CB21EZ, UK; fax: (+44)1223-336-033; or deposit@ccdc.cam.ac.uk).

Table S1. Crystallographic parameters for crystal structures MFM-221 and MFM-222

Identification code	MFM-221	MFM-222
Empirical formula	$C_{45}H_{49}Bi_2N_5O_{19}$	$C_{35}H_{23}Bi_2NO_{18}$
Formula weight	1381.85	1163.50
Temperature/K	100.15	100.00(10)
Crystal system	monoclinic	monoclinic
Space group	C2/c	Ia
a/Å	25.9161(5)	19.0775(10)
b/Å	10.14340(10)	7.2177(3)
c/Å	22.2019(4)	23.0481(11)
α/°	90	90
β/°	123.381(3)	100.962(5)
γ/°	90	90
Volume/Å^3	4873.5(2)	3115.7(3)
Z	4	4
ρ_{calc}g/cm^3	1.883	2.480
μ/mm^{-1}	14.718	11.377
F(000)	2688.0	2200.0
Crystal size/mm^3	0.2 × 0.12 × 0.02	0.048 × 0.037 × 0.026
Radiation	CuKα (λ = 1.54184)	Mo Kα (λ = 0.71073)
2Θ range for data collection/°	9.698 to 152.51	3.6 to 52.728
Index ranges	$-31 \le h \le 32$, $-10 \le k \le 12$, $-24 \le l \le 26$	$-23 \le h \le 23$, $-9 \le k \le 9$, $-28 \le l \le 28$
Reflections collected	15224	18366
Independent reflections	4842 [R_{int} = 0.0386, R_{sigma} = 0.0318]	6121 [R_{int} = 0.0696, R_{sigma} = 0.0825]
Data/restraints/parameters	4842/60/345	6121/780/514
Goodness-of-fit on F^2	1.079	1.044
Final R indexes [I>=2σ (I)]	R_1 = 0.0396, wR_2 = 0.1117	R_1 = 0.0453, wR_2 = 0.1024
Final R indexes [all data]	R_1 = 0.0410, wR_2 = 0.1134	R_1 = 0.0565, wR_2 = 0.1068
Largest diff. peak/hole / e Å^{-3}	2.12/-2.57	5.11/-1.85

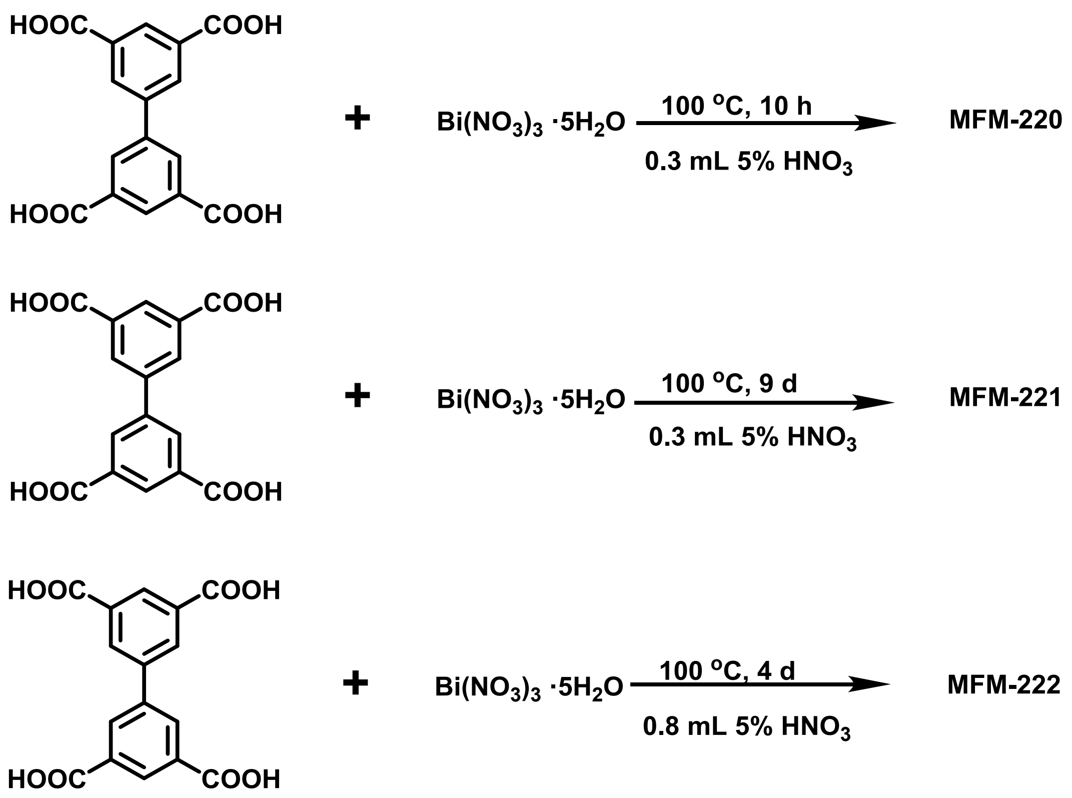

Figure S1. Synthesis of single crystals of MFM-220, MFM-221 and MFM-222.

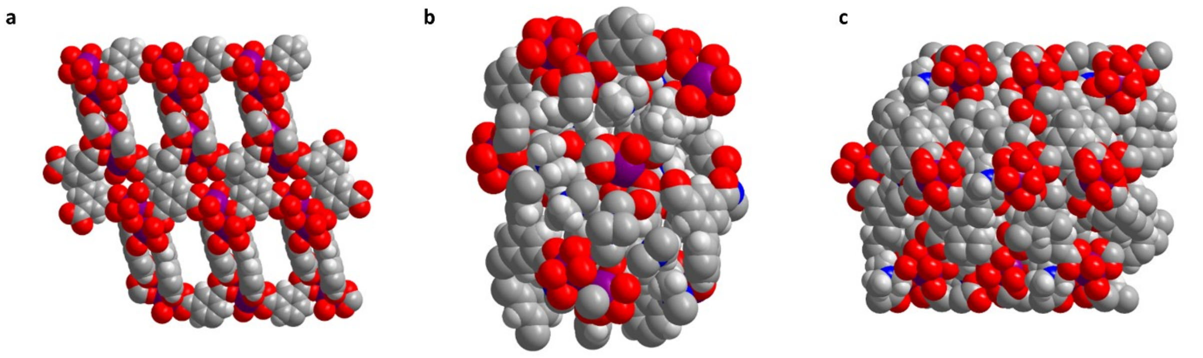

Figure S2. Space-filling representation of the structures of (a) MFM-220, (b) MFM-221 and (c) MFM-222. Colour code for atoms: Bi, purple; O, red; C, grey; H, light grey; N, blue.

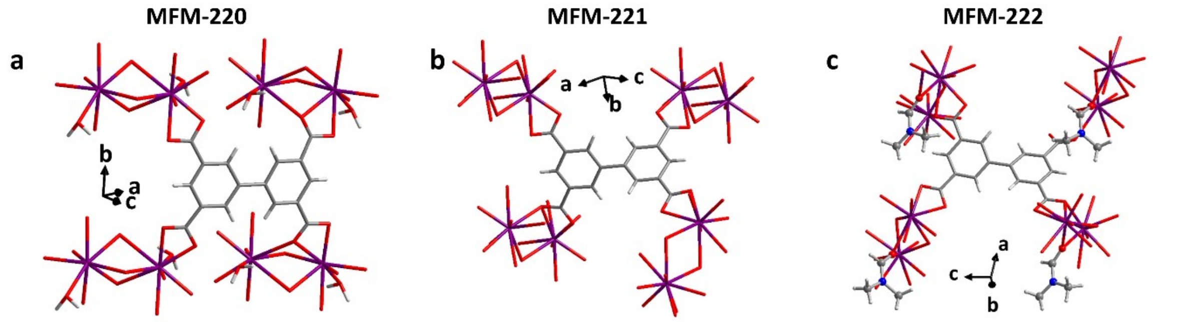

Figure S3. Views of part structures of MFM-220, MFM-221 and MFM-222. Colour code for atoms: Bi, purple; O, red; C, grey; H, light grey; N, blue.

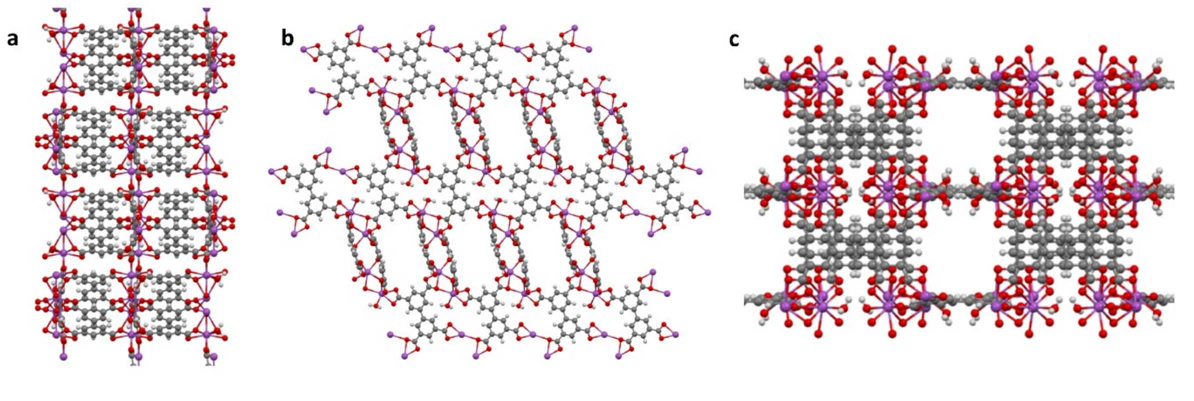

Figure S4. Views of the structure of MFM-220 along the (a) *a* axis, (b) *b* axis, and (c) *c* axis. Colour code for atoms: Bi, purple; O, red; C, grey; H, light grey; N, blue.

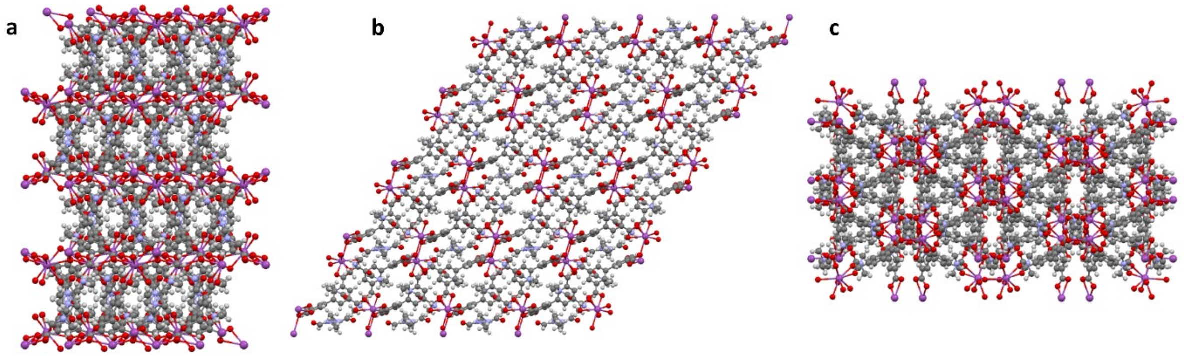

Figure S5. Views of the structure of MFM-221 along the (a) *a* axis, (b) *b* axis, and (c) *c* axis. Colour code for atoms: Bi, purple; O, red; C, grey; H, light grey; N, blue.

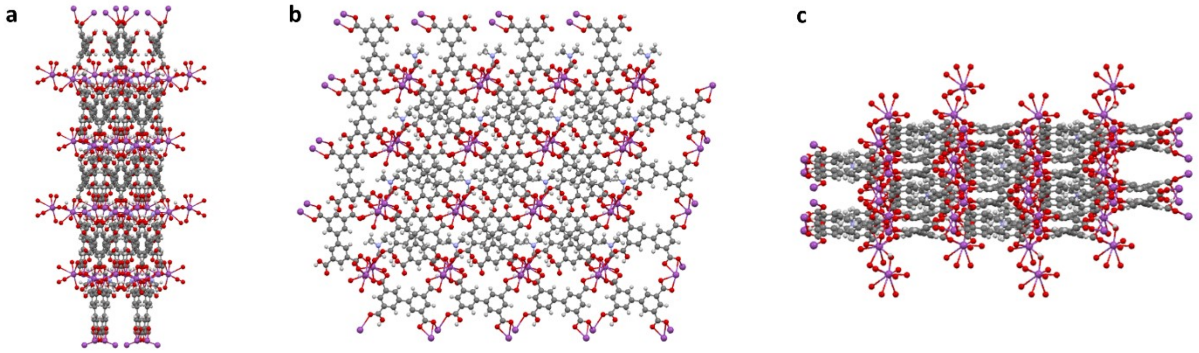

Figure S6. Views of the structure of MFM-222 along the (a) *a* axis, (b) *b* axis, and (c) *c* axis. Colour code for atoms: Bi, purple; O, red; C, grey; H, light grey; N, blue.

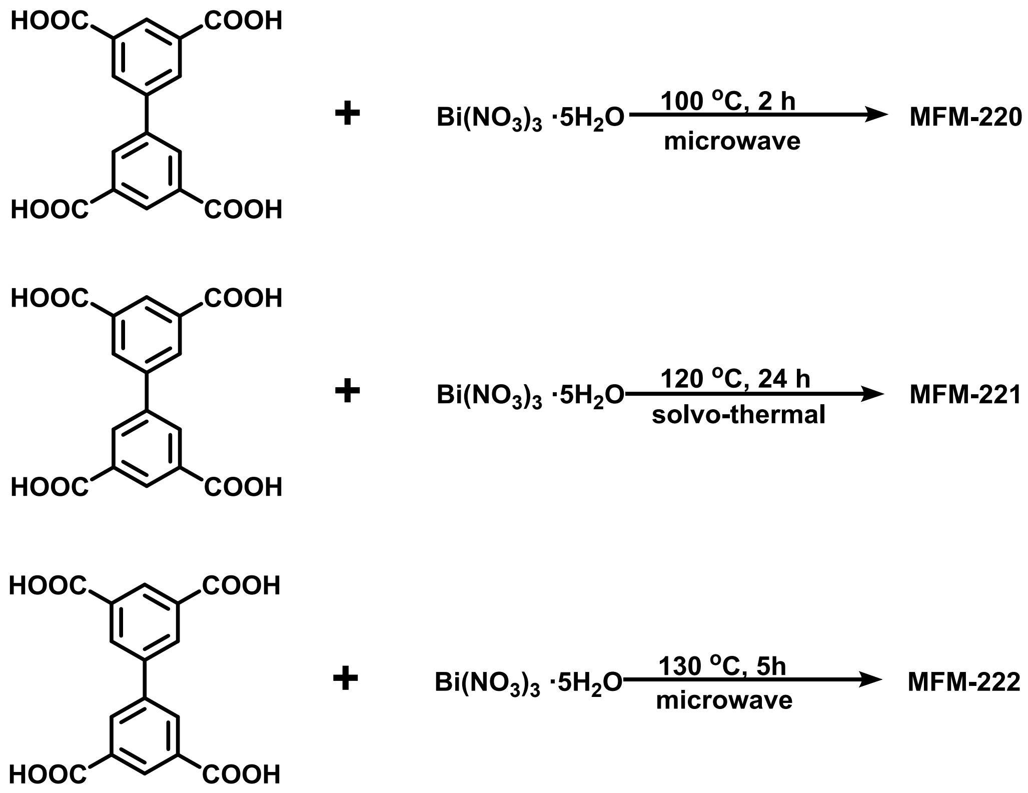

Figure S7. Route to synthesis of bulk MFM-220, MFM-221 and MFM-222.

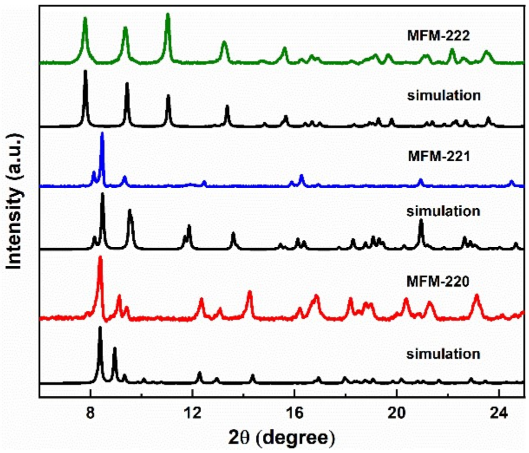

Figure S8. PXRD patterns of MFM-220, MFM-221 and MFM-222.

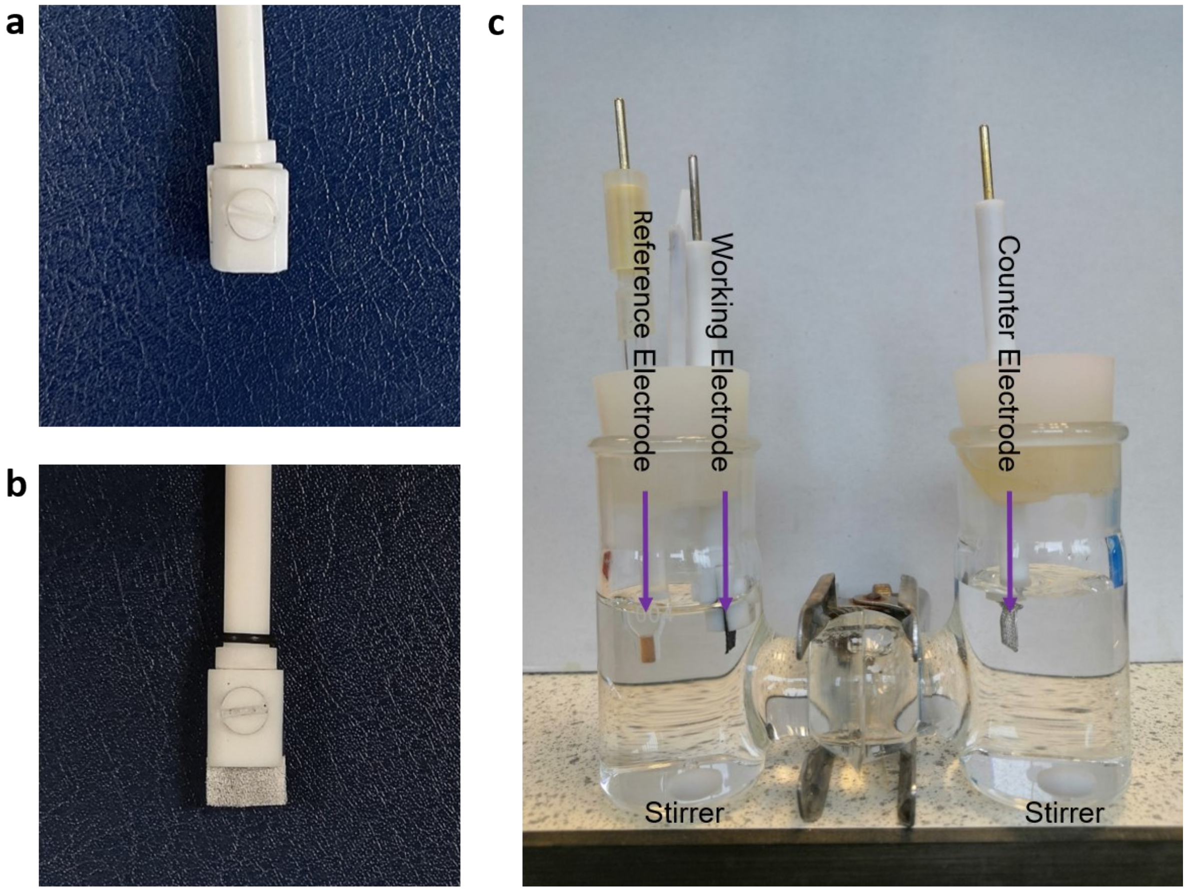

Figure S9. Views of (a) electrode holder, (b) working electrode, and (c) H-cell used in this study.

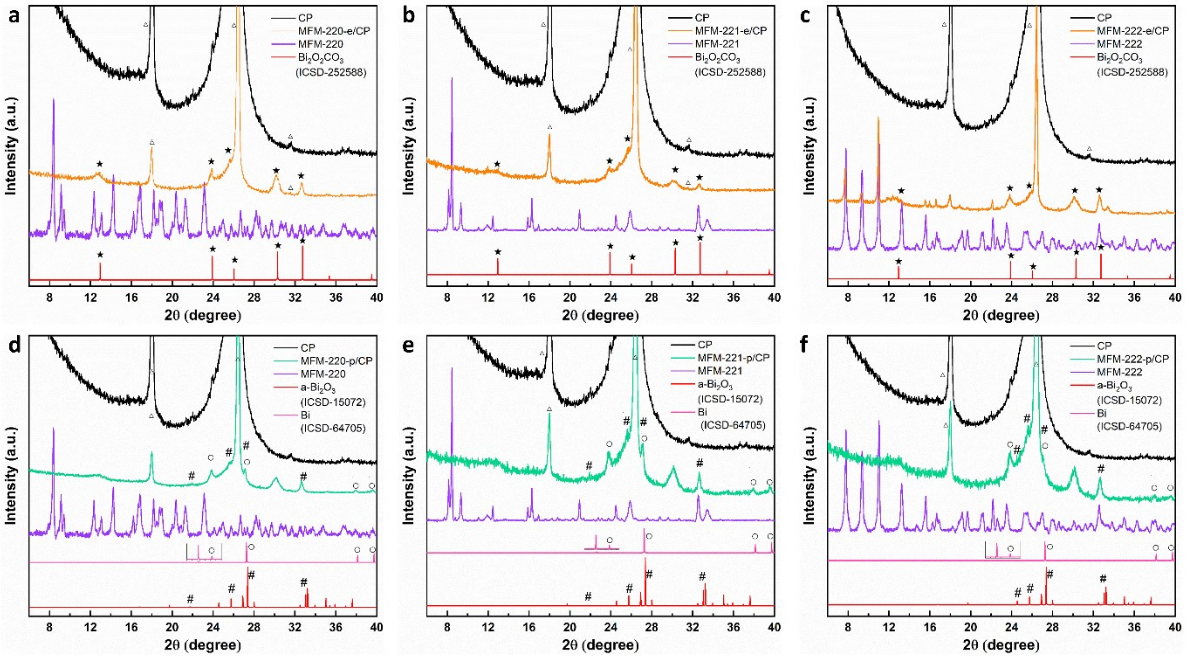

Figure S10. PXRD patterns of pristine Bi(III) MOFs, the electrolyte-mediated Bi-MOF-e/CP and the potential-mediated Bi-MOF-p/CP. (△ refers to the signal for CP, ★ refers to the signal for BiO_2CO_3, ＃ refers to the signal for Bi_2O_3, and ○ refers to the signal for Bi.)

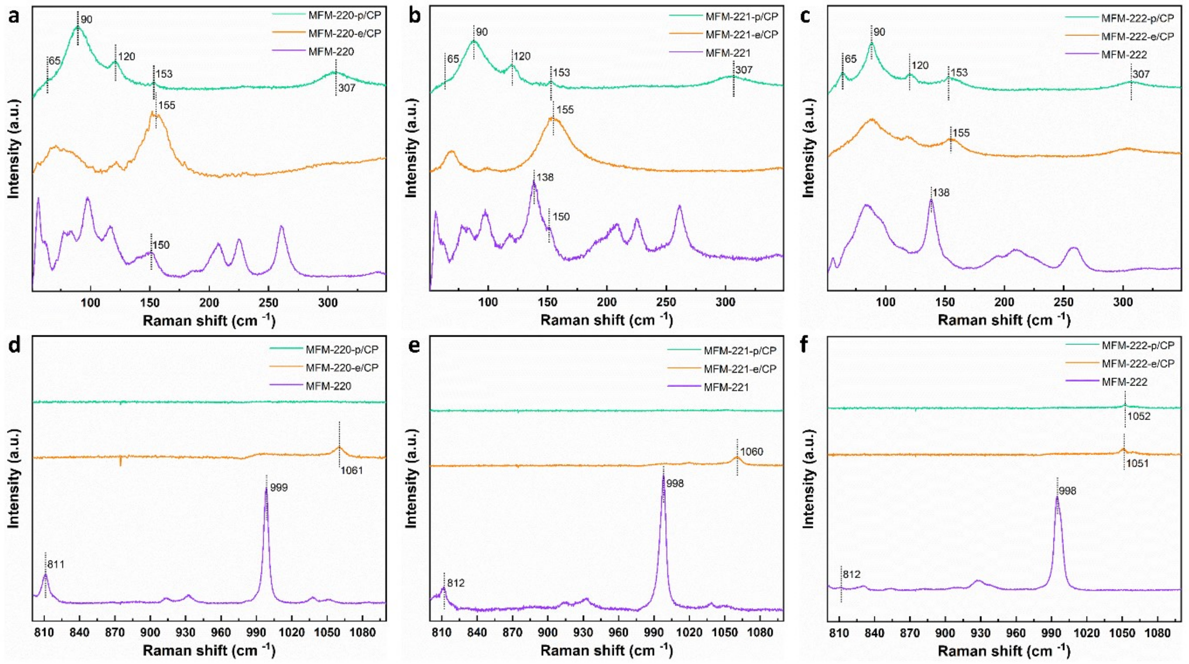

Figure S11. Raman spectra. (a,d) MFM-220, MFM-220-e/CP and MFM-220-p/CP. (b,e) MFM-221, MFM-221-e/CP and MFM-221-p/CP. (c,f) MFM-222, MFM-222-e/CP and MFM-222-p/CP.

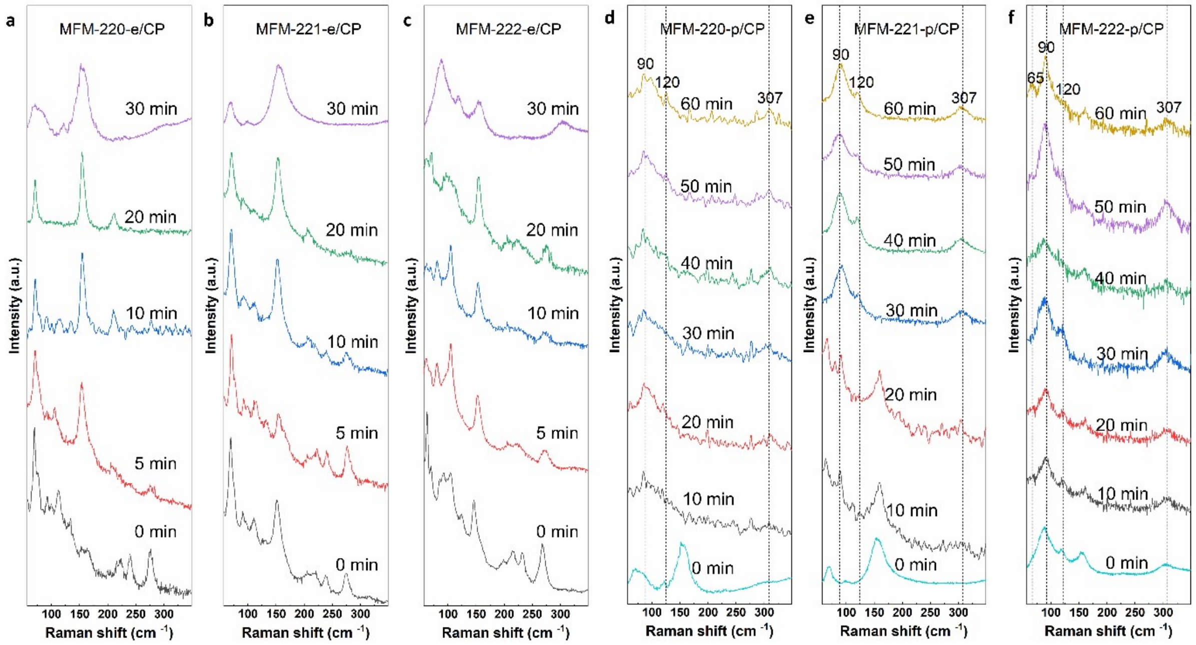

Figure S12. Raman spectroscopy monitoring the conversion of pristine MOF (0 min) to MOF-e/CP upon soaking in 0.1 M $KHCO_3$: (a) MFM-220-e/CP, (b) MFM-221-e/CP and (c) MFM-222-e/CP. Raman spectroscopy monitoring the conversion of MOF-e/CP (0 min) to MOF-p/CP upon applying reductive potential: (d) MFM-220-p/CP, (e) MFM-221-p/CP and (f) MFM-222-p/CP.

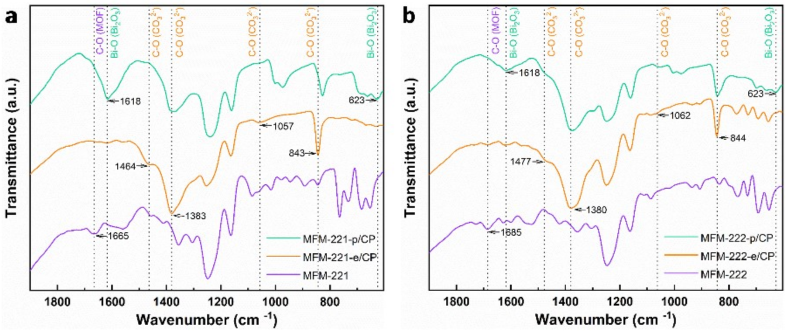

Figure S13. FT-IR spectra for (a) MFM-221, MFM-221-e/CP and MFM-221-p/CP, and for (b) MFM-222, MFM-222-e/CP and MFM-222-p/CP.

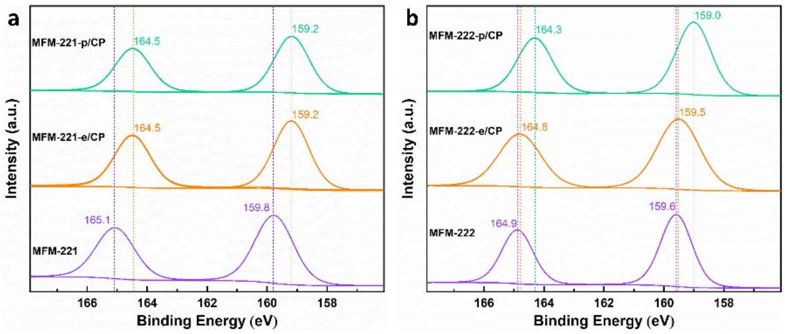

Figure S14. XPS spectra for (a) MFM-221, MFM-221-e/CP and MFM-221-p/CP, and for (b) MFM-222, MFM-222-e/CP and MFM-222-p/CP.

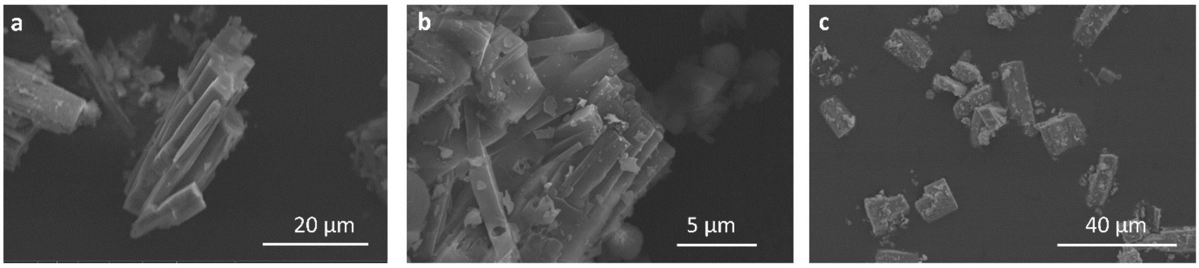

Figure S15. SEM images for (a) MFM-220, (b) MFM-221 and for (c) MFM-222.

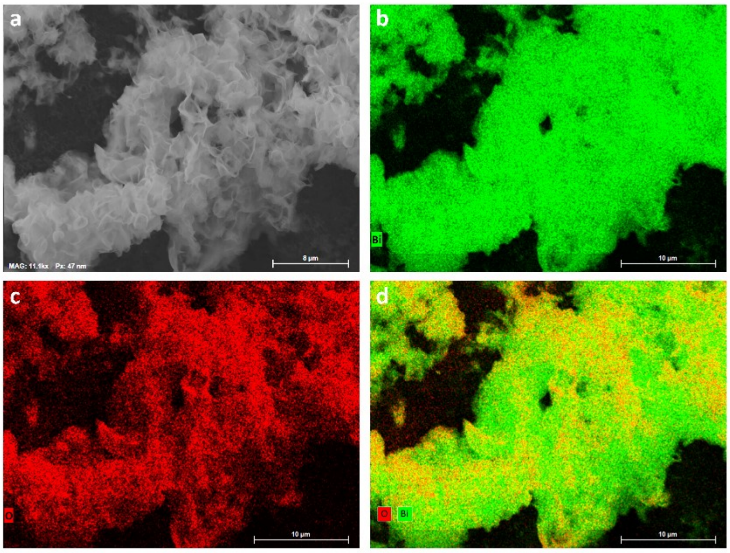

Figure S16. SEM image and SEM-EDX mapping highlighting the distribution of elements in MFM-220-e/CP. (a) SEM image, (b) distribution of Bi, (c) distribution of O. (d) Overlapping of signals due to Bi and O. Bi in green and O in red.

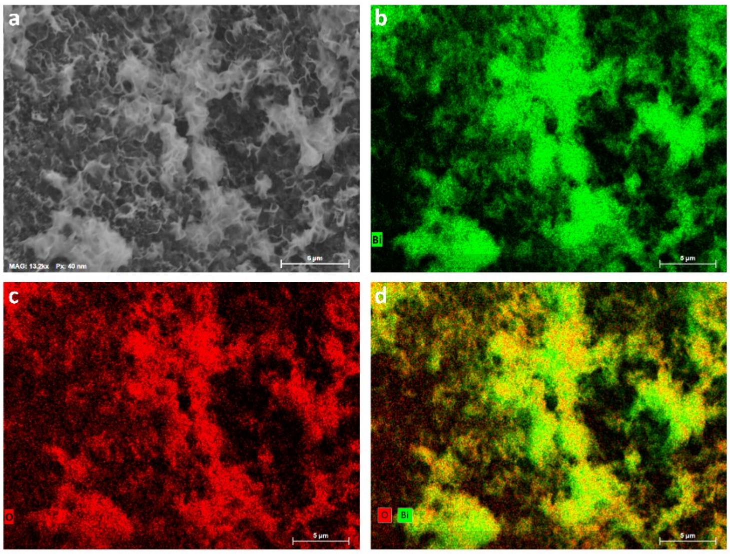

Figure S17. SEM image and SEM-EDX mapping highlighting the distribution of elements in MFM-220-p/CP. (a) SEM image, (b) distribution of Bi, (c) distribution of O. (d) Overlapping of signals due to Bi and O. Bi in green and O in red.

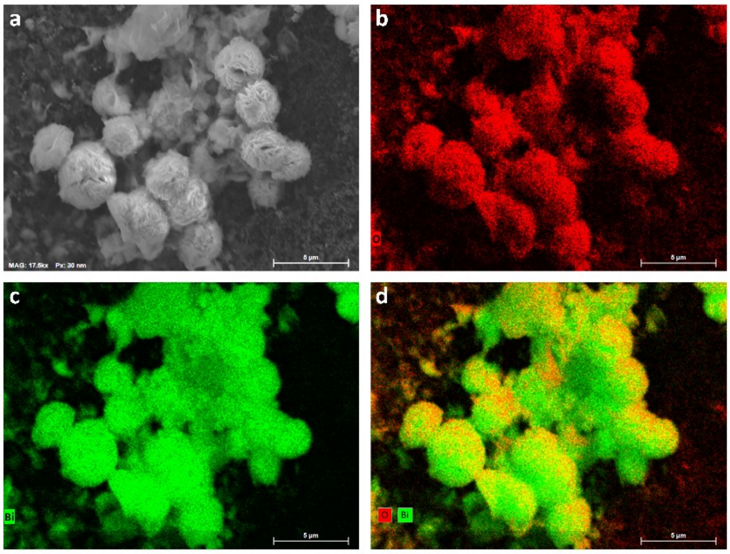

Figure S18. SEM image and SEM-EDX mapping highlighting the distribution of elements in MFM-221-e/CP. (a) SEM image, (b) distribution of Bi, (c) distribution of O. (d) Overlapping of signals due to Bi and O. Bi in green and O in red.

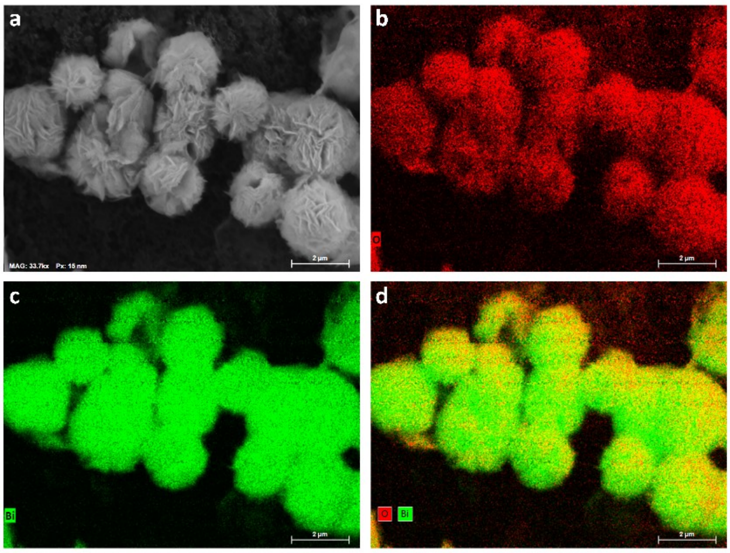

Figure S19. SEM image and SEM-EDX mapping highlighting the distribution of elements in MFM-221-p/CP. (a) SEM image, (b) distribution of Bi, (c) distribution of O. (d) Overlapping of signals due to Bi and O. Bi in green and O in red.

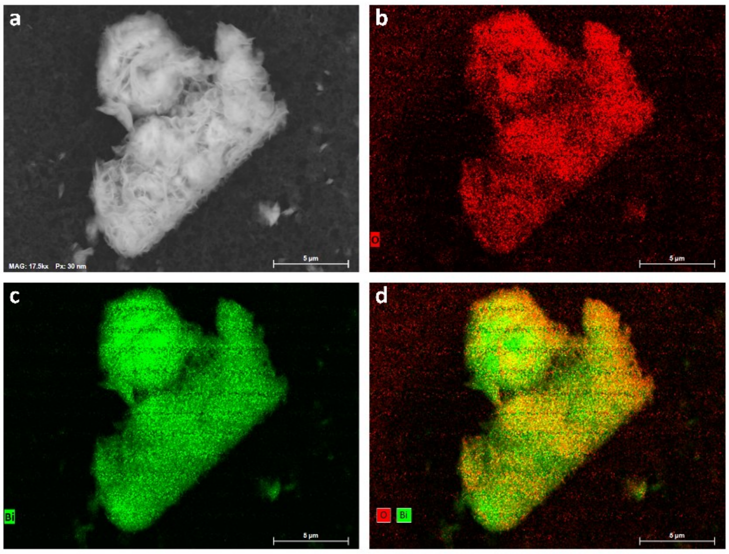

Figure S20. SEM image and SEM-EDX mapping highlighting the distribution of elements in MFM-222-e/CP. (a) SEM image, (b) distribution of Bi, (c) distribution of O. (d) Overlapping of signals due to Bi and O. Bi in green and O in red.

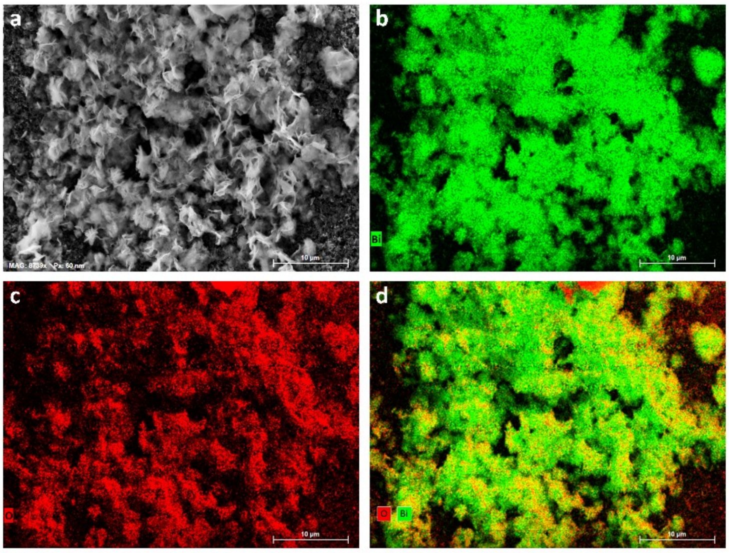

Figure S21. SEM image and SEM-EDX mapping highlighting the distribution of elements in MFM-222-p/CP. (a) SEM image, (b) distribution of Bi, (c) distribution of O. (d) Overlapping of signals due to Bi and O. Bi in green and O in red.

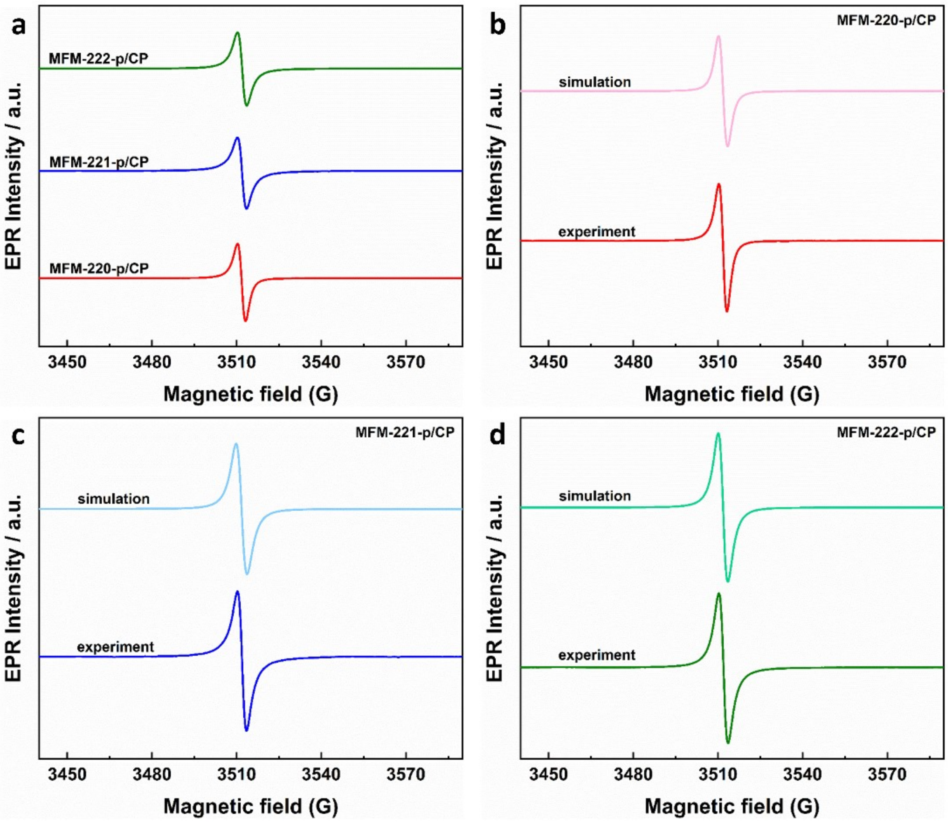

Figure S22. EPR spectra of MFM-220-p/CP, MFM-221-p/CP, MFM-222-p/CP.

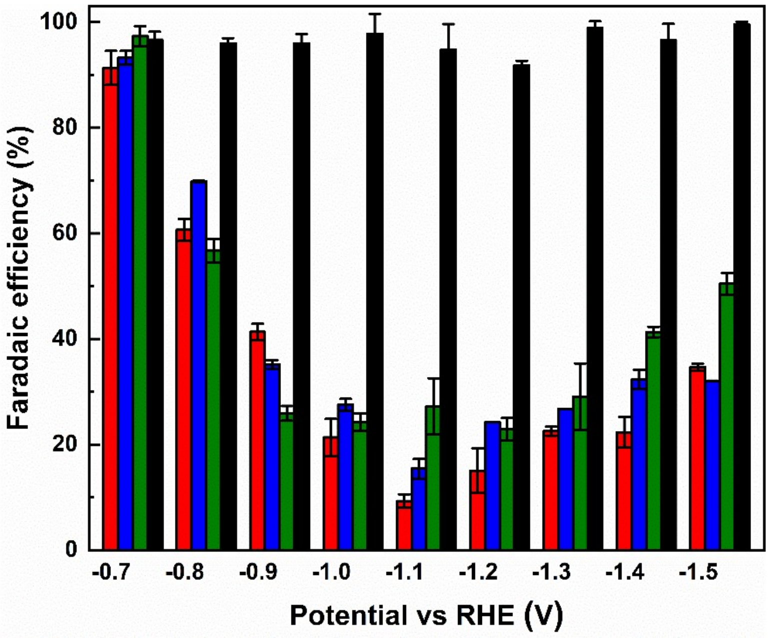

Figure S23. Plot of FE_{H2} vs potential for reduction of CO_2 using MFM-220-p/CP (red), MFM-221-p/CP (blue), MFM-222-p/CP (green) and CP (black) electrodes in 0.1 M $KHCO_3$.

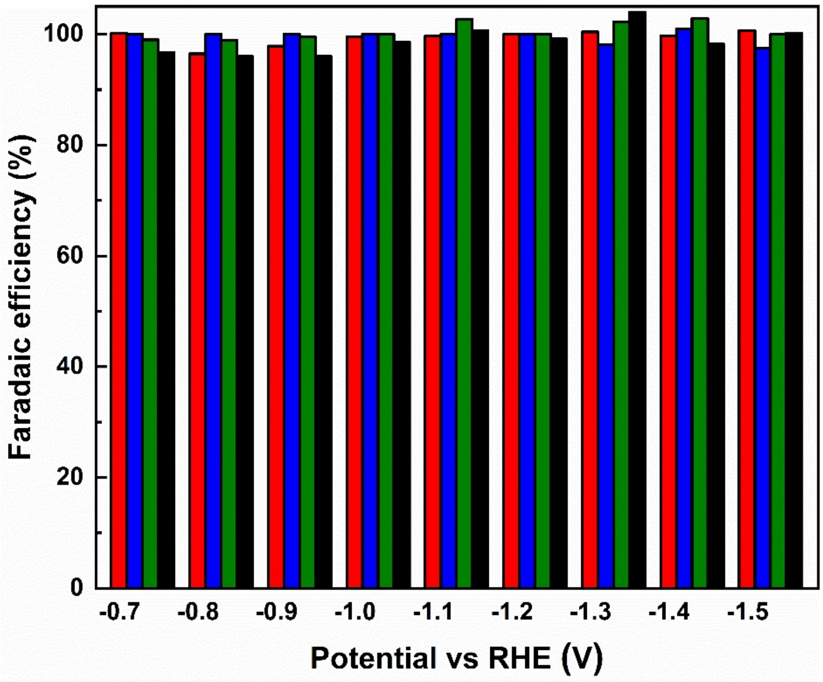

Figure S24. Plot of $FE_{formate+H2}$ vs potential for reduction of CO_2 using MFM-220-p/CP (red), MFM-221-p/CP (blue), MFM-222-p/CP (green) and CP (black) electrodes in 0.1 M $KHCO_3$.

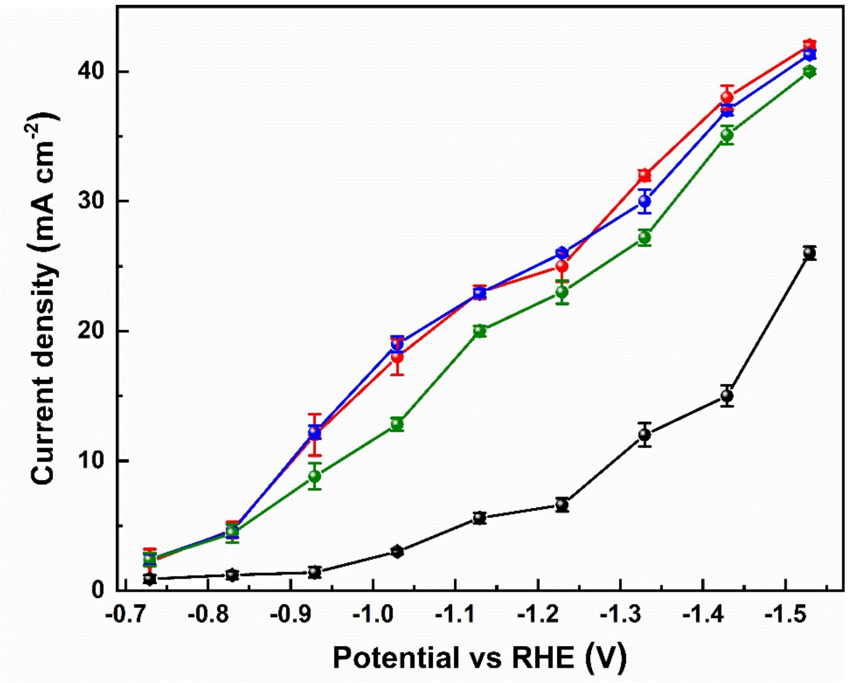

Figure S25. Plot of total current density vs potential for reduction of CO_2 using MFM-220-p/CP (red), MFM-221-p/CP (blue), MFM-222-p/CP (green) and CP (black) electrodes in 0.1 M $KHCO_3$.

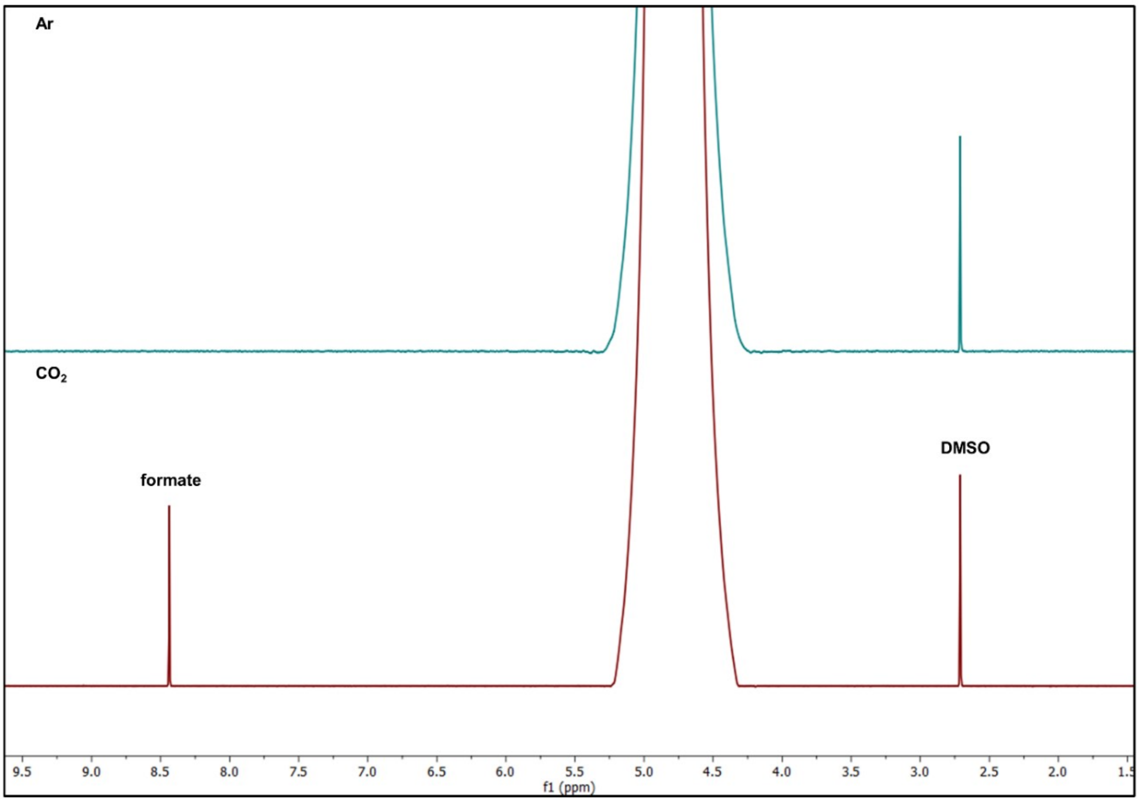

Figure S26. ^{1}H NMR spectra of liquid product in Ar-saturated and CO_2-saturated electrolyte.

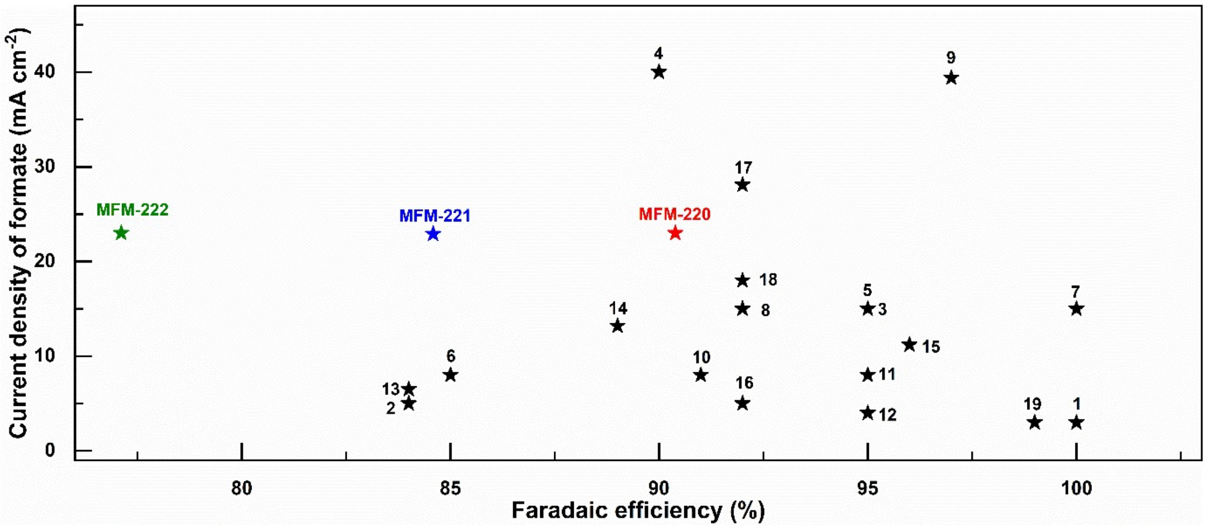

Figure S27. Comparison of literature data for reduction of CO_2 using Bi-based catalysts, the details of which are listed in Table S2).

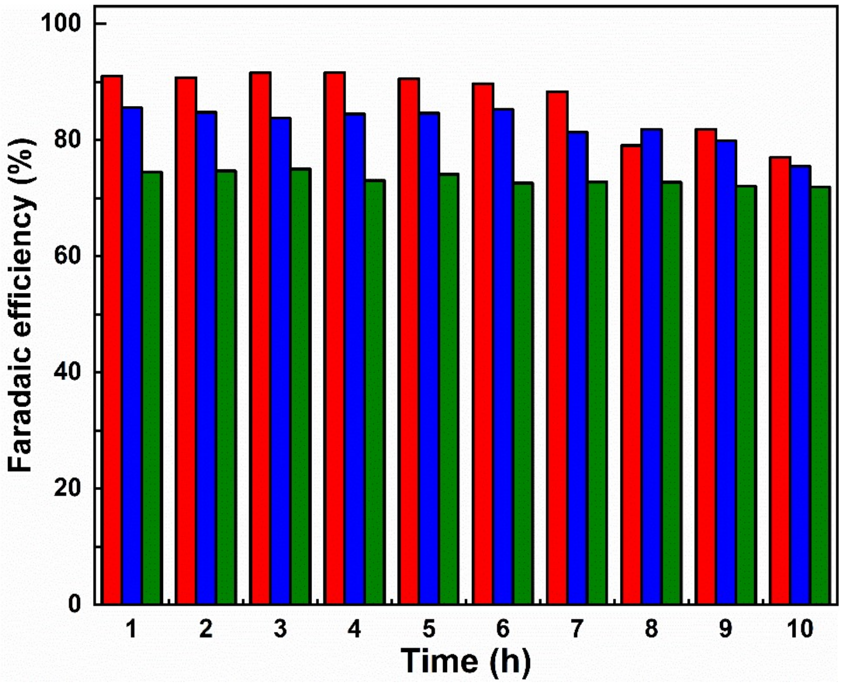

Figure S28. Plot of $FE_{formate}$ vs time for reduction of CO_2 using MFM-220-p/CP (red), MFM-221-p/CP (blue), and MFM-222-p/CP (green) electrodes in 0.1 M $KHCO_3$.

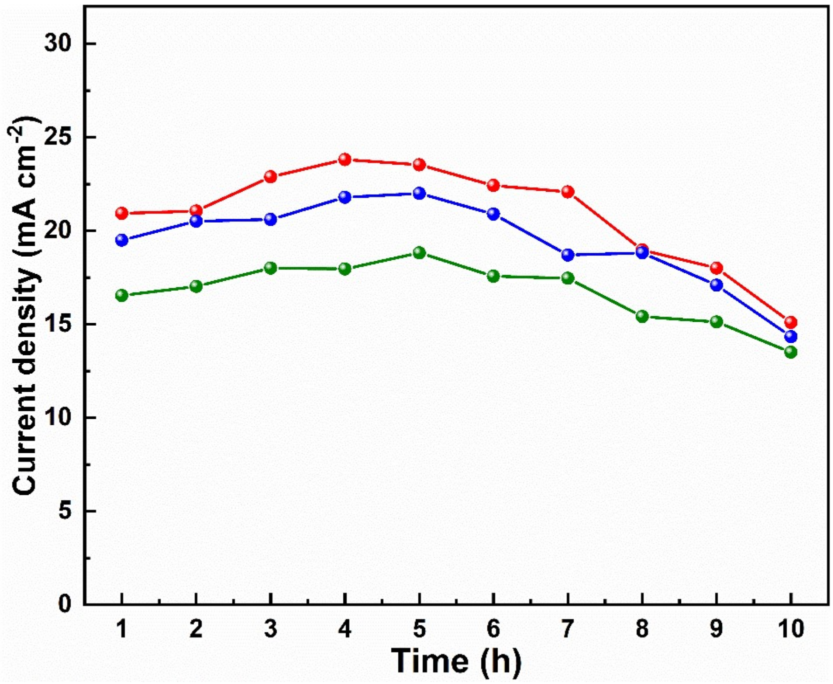

Figure S29. Plot of current density for formate production vs time for reduction of CO_2 using MFM-220-p/CP (red), MFM-221-p/CP (blue), and MFM-222-p/CP (green) electrodes in 0.1 M $KHCO_3$.

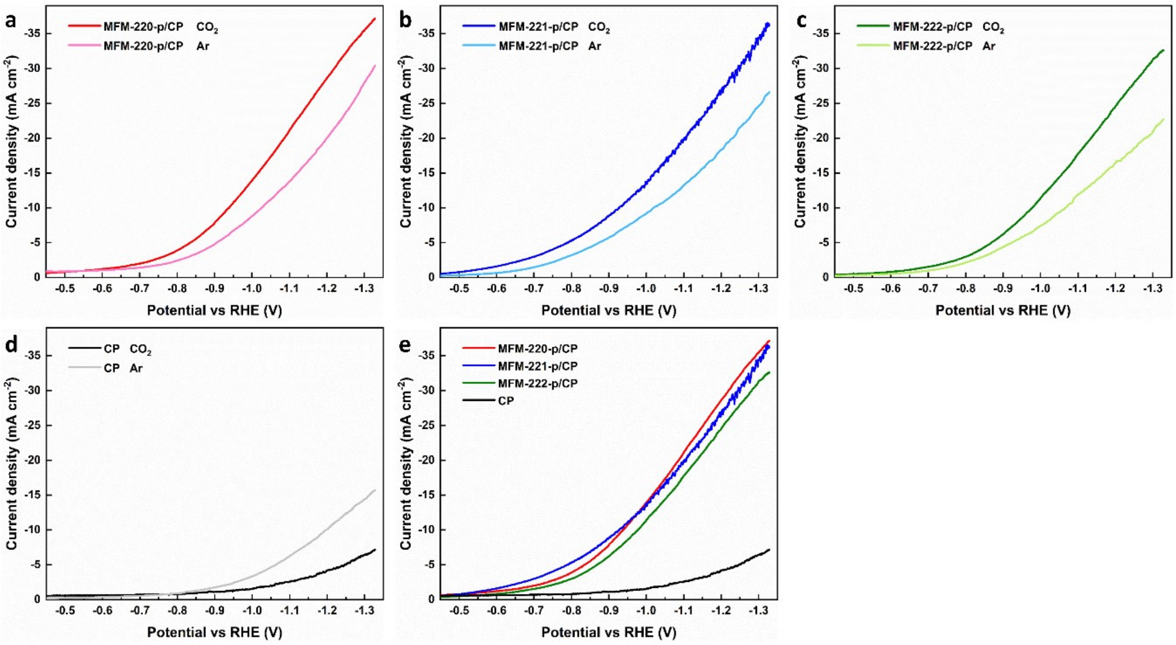

Figure S30. Linear sweep voltammetry of CO_2-saturated and Ar-saturated electrolyte at MFM-220-p/CP, MFM-221-p/CP, MFM-222-p/CP and CP electrodes.

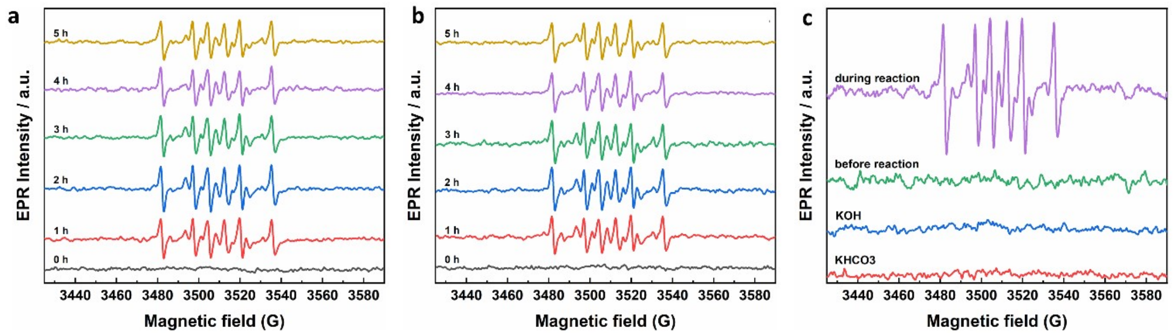

Figure S31. EPR spectra acquired at different intervals of time using Bi-MOFs-p/CP as catalyst at −1.1 V vs RHE. (a) MFM-221-p/CP, and (b) MFM-222-p/CP. (c) Comparisons of X-band EPR spectra of 0.1 M KOH, 0.1 M $KHCO_3$ electrolyte and EPR spectra before and during electrolysis. The electrolysis was carried out at −1.1 V vs RHE in 0.1 M $KHCO_3$.

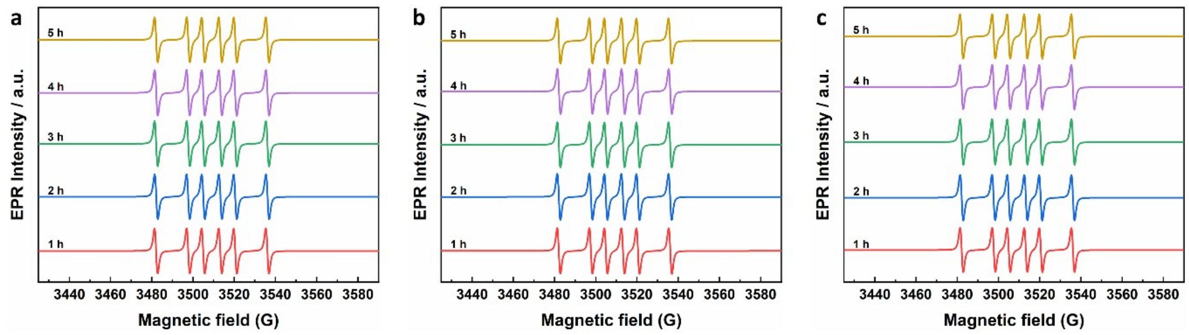

Figure S32. Simulation of change in signal for DMPO-·COOH over time using Bi-MOFs-p/CP as catalyst at −1.1 V vs RHE. (a) MFM-220-p/CP, (b) MFM-221-p/CP, and (c) MFM-222-p/CP.

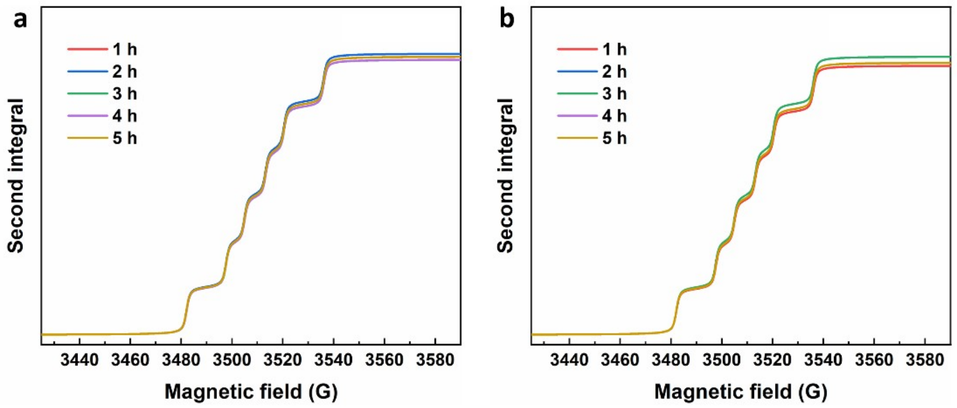

Figure S33. Second integral of the change in signal for DMPO-·COOH over time using Bi-MOFs-p/CP as catalyst at −1.1 V vs RHE. (a) MFM-221-p/CP, and (b) MFM-222-p/CP.

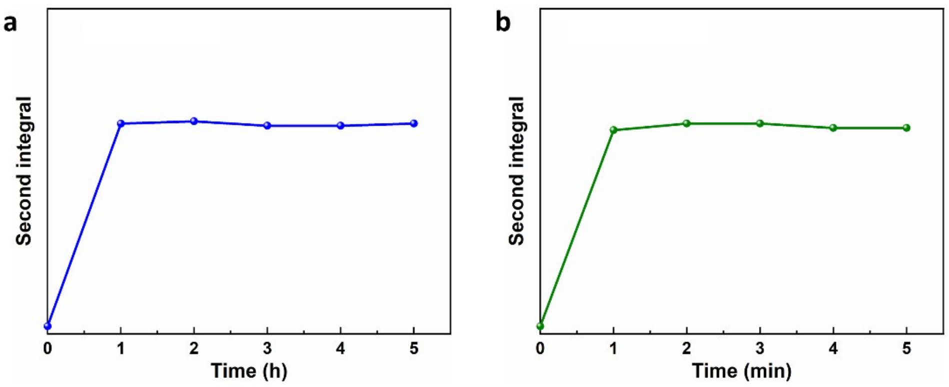

Figure S34. Plot of the second integral of the X-band EPR signals for DMPO-·COOH at room temperature vs. time of electrolysis of CO_2. (a) MFM-221-p/CP, and (b) MFM-222-p/CP.

Table S2. The catalytic performance for electrochemical reduction of CO_2 to formate using different cathodes in aqueous electrolyte in an H-cell.

Number of electrode	Electrode	electrolyte	Potential	Current density mA/cm^{-2}	FE	ref
1	Bi nanoflakes	0.1 M $KHCO_3$	-0.6 V_{RHE}	3	100%	4
2	Bi_2S_3	0.5 M $NaHCO_3$	-0.75 V_{RHE}	5	84%	5
3	Bi nanosheet	0.5 M $NaHCO_3$	-1.5 V_{SCE}	15	95%	6
4	$Bi_2O_2CO_3$	0.5 M $NaHCO_3$	-1.2 V_{RHE}	40	90%	7
5	Cu-Bi	0.5 M $NaHCO_3$	-0.93 V_{RHE}	15	95%	8
6	Bi nanosheet morphology	0.1 M $KHCO_3$	-0.85 V_{RHE}	8	85%	9
7	Bi nanoparticles	0.5 M $KHCO_3$	-1.5 V vs AgCl/Ag	15	100%	10
8	Bi nanostructure	0.5 M $KHCO_3$	- 0.9 V_{RHE}	15	92%	11
9	Bismuth Nanotubes	0.5 M $KHCO_3$	-1.1 V_{RHE}	39.4	97%	12
10	Bi oxides	0.5 M $KHCO_3$	-0.9 V_{RHE}	8	91%	13
11	Bi NP@MWCNTs	0.5 M $KHCO_3$	-1.5 V_{SCE}	8	95%	14
12	Bi(btb)	0.5 M $KHCO_3$	−0.97 V_{RHE}	4	95%	15
13	BiOCl NC	0.5 M $KHCO_3$	- 0.87 V_{RHE}	6.5	84%	16
14	$Bi_7O_9I_3$	0.5 M $KHCO_3$	0.89 V_{RHE}	13.2	89%	17
15	Bi-BTC-D	0.5 M $KHCO_3$	- 0.86 V_{RHE}	11.2	96 %	18
16	CAU-17	0.1 $KHCO_3$	-0.9 V_{RHE}	5	92%	19
17	Bi/Gr catalysts	0.5 M $KHCO_3$	-0.97 V_{RHE}	28.1	92 %	20
18	Bi-MWCNT-COOH/Cu	0.5 M $KHCO_3$	- 0.76 V_{RHE}	18	92 %	21
19	Bi monolayer	0.5 M $KHCO_3$	-0.58 V_{RHE}	3	99%	22

Table S3. EPR spectra simulation parameters of the radicals produced during CO_2RR.

Adduct	g-factor	$A^{14}N$ / G	$A\ ^1H$ / G	lw/mT
DMPO-COOH	2.0055	15.6	22.9	0.12
				0.20
DMPO-OH	2.0056	14.7	14.7	0.12
				0.09
Oxidised DMPO	2.0055	15.1	-	0.14

* lw is the homogeneous Lorentzian linewidth; *g* and *A* are the g and hyperfine constant parameters, respectively.

Reference

1. Lin, X.; Telepeni, I.; Blake, A. J.; Dailly, A.; Brown, C. M.; Simmons, J. M.; Zoppi, M.; Walker, G. S.; Thomas, K. M.; Mays, T. J., High capacity hydrogen adsorption in Cu (II) tetracarboxylate framework materials: the role of pore size, ligand functionalisation, and exposed metal sites. *J. Am. Chem. Soc.* **2009**, *131*, 2159-2171.
2. Stoll, S. & Schweiger, A. EasySpin, a comprehensive software package for spectral simulation and analysis in EPR. *J. Magn. Reson.* **2006**, *178*, 42-55.
3. a) Sheldrick, G. M. Crystal structure refinement with SHELXL. Acta Crystallogr. Sect. C Struct. Chem. **2015**, 71, 3–8. b)Dolomanov, O. V., Bourhis, L. J., Gildea, R. J., Howard, J. A. K. & Puschmann, H. OLEX2 : a complete structure solution, refinement and analysis program. J. Appl. Crystallogr. **2009**, *42*, 339–341.
4. Kim, S.; Dong, W. J.; Gim, S.; Sohn, W.; Park, J. Y.; Yoo, C. J.; Jang, H. W.; Lee, J. L., Shape-controlled bismuth nanoflakes as highly selective catalysts for electrochemical carbon dioxide reduction to formate. *Nano Energy* **2017**, *39*, 44-52.
5. Zhang, Y.; Li, F. W.; Zhang, X. L.; Williams, T.; Easton, C. D.; Bond, A. M.; Zhang, J., Electrochemical reduction of CO_2 on defect-rich Bi derived from Bi_2S_3 with enhanced formate selectivity. *J. Mater. Chem. A* **2018**, *6*, 4714-4720.
6. Han, N.; Wang, Y.; Yang, H.; Deng, J.; Wu, J. H.; Li, Y. F.; Li, Y. G., Ultrathin bismuth nanosheets from in situ topotactic transformation for selective electrocatalytic CO_2 reduction to formate. *Nat. Commun.* **2018**, *9*, 1-8.
7. Liu, P. F.; Zu, M. Y.; Zheng, L.; Yang, H. G., Bismuth oxyiodide microflower-derived catalysts for efficient CO_2 electroreduction in a wide negative potential region. *Chem. Commun.* **2019**, *55*, 12392-12395.
8. Jia, L.; Yang, H.; Deng, J.; Chen, J. M.; Zhou, Y.; Ding, P.; Li, L. G.; Han, N.; Li, Y. G., Copper-bismuth bimetallic microspheres for selective electrocatalytic reduction of CO_2 to formate. *Chin. J. Chem.* **2019**, *37*, 497-500.
9. Gao, T. F.; Wen, X. M.; Xie, T. H.; Han, N. N.; Sun, K.; Han, L.; Wang, H. C.; Zhang, Y.; Kuang, Y.; Sun, X. M., Morphology effects of bismuth catalysts on electroreduction of carbon dioxide into formate. *Electrochim. Acta* **2019**, *305*, 388-393.
10. Avila-Bolivar, B.; Garcia-Cruz, L.; Montiel, V.; Solla-Gullon, J., Electrochemical Reduction of CO_2 to formate on easily prepared carbon-supported Bi nanoparticles. *Molecules* **2019**, *24*, 2032.
11. Lu, P. L.; Gao, D. L.; He, H. Y.; Wang, Q. X.; Liu, Z. J.; Dipazir, S.; Yuan, M. T.; Zu, W. Y.; Zhang, G. J., Facile synthesis of a bismuth nanostructure with enhanced selectivity for electrochemical conversion of CO_2 to formate. *Nanoscale* **2019**, *11*, 7805-7812.
12. Fan, K.; Jia, Y. F.; Ji, Y. F.; Kuang, P. Y.; Zhu, B. C.; Liu, X. Y.; Yu, J. G., Curved Surface Boosts Electrochemical CO_2 Reduction to formate via bismuth nanotubes in a wide potential window. *ACS Catal.* **2020**, *10*, 358-364.
13. Deng, P. L.; Wang, H. M.; Qi, R. J.; Zhu, J. X.; Chen, S. H.; Yang, F.; Zhou, L.; Qi, K.; Liu, H. F.; Xia, B. Y., Bismuth oxides with enhanced bismuth-oxygen structure for efficient electrochemical reduction of carbon dioxide to formate. *ACS Catal.* **2020**, *10*, 743-750.

14. Zhang, X.; Fu, J.; Liu, Y. Y.; Zhou, X. D.; Qiao, J. L., Bismuth Anchored on MWCNTs with Controlled Ultrafine Nanosize Enables High-Efficient Electrochemical reduction of carbon dioxide to formate fuel. *ACS Sustain. Chem. Eng.* **2020**, *8*, 4871-4876.

15. Lamagni, P.; Miola, M.; Catalano, J.; Hvid, M. S.; Mamakhel, M. A. H.; Christensen, M.; Madsen, M. R.; Jeppesen, H. S.; Hu, X. M.; Daasbjerg, K.; Skrydstrup, T.; Lock, N., Restructuring metal-organic frameworks to nanoscale bismuth electrocatalysts for highly active and selective CO_2 reduction to formate. *Adv. Funct. Mater.* **2020**, *30*, 1910408.

16. Barik, R.; Subramanian, S.; Chukwuike, V.; AnbuKulandainathan, M., Bismuth oxychloridedispersed on nitrogen doped carbon as a catalyst for the electrochemical conversion of CO_2 to formate. *ChemElectroChem* **2020**, *7*, 2265-2273.

17. Wang, Q.; Ma, M.; Zhang, S.; Lu, K.; Fu, L.; Liu, X.; Chen, Y., Influence of the chemical compositions of bismuth oxyiodides on the electroreduction of carbon dioxide to formate. *ChemPlusChem* **2020**, *85*, 672-678.

18. Zhang, X.; Zhang, Y.; Li, Q.; Zhou, X.; Li, Q.; Yi, J.; Liu, Y.; Zhang, J., Highly efficient and durable aqueous electrocatalytic reduction of CO_2 to HCOOH with a novel bismuth–MOF: experimental and DFT studies. *J. Mater. Chem. A* **2020**, *8*, 9776-9787.

19. Li, F.; Gu, G. H.; Choi, C.; Kolla, P.; Hong, S.; Wu, T.-S.; Soo, Y.-L.; Masa, J.; Mukerjee, S.; Jung, Y., Highly stable two-dimensional bismuth metal-organic frameworks for efficient electrochemical reduction of CO_2. *Appl. Catal. B* **2020**, *277*, 119241.

20. Wu, D.; Chen, W.; Wang, X.; Fu, X.-Z.; Luo, J.-L., Metal-support interaction enhanced electrochemical reduction of CO_2 to formate between graphene and Bi nanoparticles. *J. CO_2 Util.* **2020**, *37*, 353-359.

21. Li, Q.; Zhang, X.; Zhou, X.; Li, Q.; Wang, H.; Yi, J.; Liu, Y.; Zhang, J., Simply and effectively electrodepositing Bi-MWCNT-COOH composite on Cu electrode for efficient electrocatalytic CO_2 reduction to produce HCOOH. *J. CO_2 Util.* **2020**, *37*, 106-112.

22. Yang, F.; Elnabawy, A. O.; Schimmenti, R.; Song, P.; Wang, J.; Peng, Z.; Yao, S.; Deng, R.; Song, S.; Lin, Y., Bismuthene for highly efficient carbon dioxide electroreduction reaction. *Nat. Commun.* **2020**, *11*, 1088.
